# Advances in the development of *Salmonella*‐based vaccine strategies for protection against Salmonellosis in humans

**DOI:** 10.1111/jam.15055

**Published:** 2021-04-03

**Authors:** K.T. Sears, J.E. Galen, S.M. Tennant

**Affiliations:** ^1^ Center for Vaccine Development and Global Health Department of Medicine University of Maryland School of Medicine Baltimore MD USA

## Abstract

*Salmonella* spp. are important human pathogens globally causing millions of cases of typhoid fever and non‐typhoidal salmonellosis annually. There are only a few vaccines licensed for use in humans which all target *Salmonella enterica* serovar Typhi. Vaccine development is hampered by antigenic diversity between the thousands of serovars capable of causing infection in humans. However, a number of attenuated candidate vaccine strains are currently being developed. As facultative intracellular pathogens with multiple systems for transporting effector proteins to host cells, attenuated *Salmonella* strains can also serve as ideal tools for the delivery of foreign antigens to create multivalent live carrier vaccines for simultaneous immunization against several unrelated pathogens. Further, the ease with which *Salmonella* can be genetically modified and the extensive knowledge of the virulence mechanisms of this pathogen means that this bacterium has often served as a model organism to test new approaches. In this review we focus on (1) recent advances in live attenuated *Salmonella* vaccine development, (2) improvements in expression of foreign antigens in carrier vaccines and (3) adaptation of attenuated strains as sources of purified antigens and vesicles that can be used for subunit and conjugate vaccines or together with attenuated vaccine strains in heterologous prime‐boosting immunization strategies. These advances have led to the development of new vaccines against *Salmonella* which have or will soon be tested in clinical trials.

## Introduction

*Salmonella* spp. are Gram‐negative, intracellular, facultative anaerobic and motile rods. They can be divided into two species: *Salmonella enterica* and *Salmonella bongori*. *Salmonella enterica* subspecies *enterica* is the primary species that causes infection in humans and animals. More than 2500 serovars have been identified based on their O‐polysaccharide (OPS) and flagellin (O and H, respectively) antigens (Grimont 2007). Infection typically occurs by ingestion of contaminated food or water. Typhoidal serovars are human‐restricted and cause enteric fever, whereas non‐typhoidal *Salmonella* (NTS) serovars are zoonotic and typically produce a self‐limiting gastroenteritis in humans. Certain NTS serovars also cause invasive NTS (iNTS) infections in infants and young children in sub‐Saharan Africa (Feasey *et al*. 2012). In low‐ and middle‐income countries (LMICs), typhoidal *Salmonella* is estimated to cause over 11 million infections and over 200 000 deaths every year; NTS is estimated to cause over 90 million cases and over 100 000 deaths annually (Mogasale *et al*. 2014; Majowicz *et al*. 2019).

The high morbidity and mortality due to typhoidal *Salmonella* and NTS as well as the increasing prevalence of multidrug resistant strains have spurred development of vaccines against these pathogens. The only licensed *Salmonella* vaccines are against *S*. Typhi, the primary cause of typhoid fever. This includes the orally administered live‐attenuated Ty21a vaccine which protects against *S*. Typhi and offers some cross‐protection against *S*. Paratyphi B but not *S*. Paratyphi A (Simanjuntak *et al*. 1991; Levine *et al*. 2007) and the injectable Vi capsular polysaccharide and conjugate vaccines which are highly protective against *S*. Typhi infection but offer no cross‐protection against other serovars unless these serovars also express Vi. This limited cross‐reactivity underscores the need for vaccines against NTS in humans particularly in Asia where *S*. Paratyphi A infections are common.

While antibodies to O polysaccharide and Vi capsular polysaccharide are highly protective, antibodies to proteins such as flagellin also elicit protective antibody responses. Additionally, CD4^+^ Th1 cells and cytotoxic CD8^+^ cells are important for cytokine production and clearance of persistent *Salmonella* infections (Mastroeni *et al*. 2001; Gayet *et al*. 2017; Gal‐Mor 2019). Furthermore, because live attenuated *Salmonella* vaccines stimulate humoral and cellular immune responses both systemically and at mucosal sites, many live vaccine candidates have been developed over the years. Due to the ease of genetically engineering *Salmonella*, they have been widely utilized as carriers to deliver heterologous antigens from a variety of pathogens and as a source of components for other vaccine formats.

In this review, we highlight the use of *Salmonella* as live attenuated vaccines, as live carrier vaccines to deliver foreign antigens or as reagent strains to purify components for subunit vaccines (summarized in Table 1).

**Table 1:** List of selected recombinant *Salmonella* strains developed as vaccine candidates or as reagent strains for other vaccine formats

Type of vaccine	Strain name	Characteristics	References
Live attenuated vaccines			
Enhanced for acid resistance	χ11623	*S*. Typhi Ty2 ΔP*_fur81_*::TT *araC* P*_araBAD_ fur* ΔP*_adiA276_*::TT *rhaSR* P*_rhaBAD_ adiA* Δ(P*_adiY_*‐*adiY*‐P*_adiC_*)‐*119 adiC*; expresses a rhamnose‐inducible arginine decarboxylase (Adi) acid resistance system	Brenneman *et al*. (2013)
	Ty21a‐Gad (MD297)	*S*. Typhi Ty21a with *S. flexneri gad* (glutamate decarboxylase) genes integrated into *tviD‐vexA* locus	Dharmasena *et al*. (2016a)
Reduced fecal shedding	AH9	*S*. Typhimurium IR715 *ΔshdA* mutant	Kingsley *et al*. (2003)
	AH12	*S*. Typhimurium IR715 *ΔratB* mutant	Kingsley *et al*. (2003)
	CWD9	*S*. Typhimurium 14028 *misL*::pGP704 Nal^R^ *ΔshdA*::*aph*	Dorsey *et al*. (2005)
	MFA17	*S*. Typhimurium Δ*aroA* Δ*misL*::*cat* Δ*shdA*::*aph*	Abd El Ghany *et al*. (2007)
Programmed attenuation and delayed lysis	χ8937(pYA3685)	*S*. Typhimurium UK‐1 *ΔasdA19::araC* P_BAD_ c2 ΔP*_murA7_*::*araC* P_BAD_ *murA Δ(gmd‐fcl)‐26*. Plasmid pYA3685 expresses pneumococcal surface protein A (PspA)	Kong *et al*. (2008)
Live carrier vaccines			
Expression of foreign antigens using chromosomal integration	Ty21a‐PA‐01	*S*. Typhi Ty21a expressing *Bacillus anthracis* PA83‐HlyA fusion integrated into *tviD*‐*vexA* locus	Sim *et al*. (2017)
	MD149	*S*. Typhi Ty21a with *Shigella dysenteriae* serotype 1 O‐antigen genes integrated into the *tviD‐vexA* locus (expressed from native promoter)	Dharmasena *et al*. (2016b)
	MD174	*S*. Typhi Ty21a with *S. dysenteriae* serotype 1 O‐antigen genes integrated into the *tviD‐vexA* locus (expressed from the *lpp* promoter)	Dharmasena *et al*. (2016b)
	MD194	*S*. Typhi Ty21a with *S. flexneri* 2a O‐antigen genes integrated into *tviD*‐*vexA* locus (expressed from native promoter)	Dharmasena *et al*. (2017)
	MD196	*S*. Typhi Ty21a with *S. flexneri* 3a O‐antigen genes integrated into *tviD*‐*vexA* locus (expressed from native promoter)	Dharmasena *et al*. (2017)
	Ty21a‐AR‐Ss	*S*. Typhi Ty21a with *S. sonnei* form I O‐antigen gene cluster and *gad* systems integrated into *tviD*‐*vexA* locus	Wu *et al*. (2017)
Use of secretion systems to export foreign antigens	LH1160	*S*. Typhimurium ATCC 14028 *ΔphoP ΔphoQ ΔpurB;* expresses *H. pylori ureA* and *ureB*	Angelakopoulos and Hohmann (2000)
	CKS257	*S*. Typhimurium SL1344 *ΔphoP/phoQ ΔaroA Δasd ΔstrA/strB* (pSB2131); expresses HIV Gag	Kotton *et al*. (2006)
	MvP525 (p2810)	*S*. Typhimurium NCTC 12023 *ΔhtrA* P*_sseA_*::GFP *ΔpurD* P*_sseA_*::OVA, Kan^R^ (carrier strain MvP525); pWSK29 SseF_1–263_::Llo_51–363_::HA (designated as p2810); T3SS effector SseF fused to *L. monocytogenes* Llo	Husseiny *et al*. (2007)
	MvP728 (p3635)	*S*. Typhimurium NCTC 12023 *ΔhtrA* P_*sseA*_::GFP *ΔpurD* P*_sseA_*::OVA, Kan^R^ (carrier strain MvP728); pWSK29 P_*sseA*_ *sseJ*::lisA51‐363::HA (designated as p3635); T3SS effector SseJ fused to *L. monocytogenes* LisA	Hegazy *et al*. (2012)
	SV9699 (pIZ2267)	*S*. Typhimurium 14028 *aroA551*::Tn10 Δ*aroB*::Km^R^;pWSK29‐P_*sseA*_‐SseJ‐PcrV‐FLAG; expresses *P. aeruginosa* V antigen (PcrV)	Aguilera‐Herce *et al*. (2019)
	CVD 908*ssb*‐TXSVN	*S*. Typhi Ty2 with survivin‐SseJ fusion; candidate in multiple myeloma clinical trial	ClinicalTrials.gov Identifier: NCT03762291
Recombinant *Salmonella* as reagent strains;			
As a source of antigen for conjugate vaccines	CVD 1925 (pSEC10‐*wzzB*)	*S*. Typhimurium I77 Δ*guaBA* Δ*clpP* Δ*fliD* Δ*fljB* overexpressing *wzzB*; produces medium to long chain COPS; secretes large amounts of FliC into supernatant; reagent strain for O:4 OPS and flagellin purification	Tennant *et al*. (2011); Baliban *et al*. (2017); Hegerle *et al*. (2018)
	CVD 1943	*S*. Enteritidis R11 *ΔguaBA ΔclpP ΔfliD;* secretes large amounts of FliC into supernatant; reagent strain for O:9 OPS and FliC purification	Tennant *et al*. (2011)
As a source of outer membrane vesicles	618 Δ*tolR* Δ*msbB* Δ*pagP*	*S*. Enteritidis with enhanced shedding of GMMAs due to *tolR* deletion; reduced reactogenicity due to deletion of *msbB* and *pagP*	De Benedetto *et al*. (2017)
	1418 Δ*tolR* Δ*htrB*	*S*. Typhimurium with enhanced shedding of GMMAs due to *tolR* deletion; reduced reactogenicity due to deletion of *htrB*	De Benedetto *et al*. (2017)

HIV, human immunodeficiency virus; T3SS, Type 3 Secretion System; OPS, O polysaccharide; GMMA, generalized modules for membrane antigens.

## Live attenuated *Salmonella* vaccines

Live oral attenuated *Salmonella* vaccines present several advantages over other vaccine formats. In addition to inducing local immune responses in the gut epithelium, live vaccines also stimulate systemic humoral and cellular immunity (Gayet *et al*. 2017). Oral administration also means that less hazardous waste is generated during delivery, and it also makes these vaccines easier to distribute to large populations. However, producing a live vaccine that is sufficiently attenuated and well tolerated while stimulating protective immune responses requires fine tuning to balance reactogenicity with immunogenicity (Galen and Curtiss 2014).

The only licensed live oral attenuated *S. *Typhi vaccine, Ty21a, was developed using chemical mutagenesis, and as a result, it carries multiple mutations including the deficiency in UDP‐galactose‐4‐epimerase activity for which it was selected (Germanier and Fuer 1975; Kopecko *et al*. 2009). This highlights the lack of specificity in generating mutants using this method. Ty21a is given in four doses for optimal responses and requires boosters every 5 years. The goal for newer generation vaccines is to develop candidates that are effective at a single dose while having better defined mutations. Genetic engineering allows for site‐directed mutagenesis and better characterization of mutants. Furthermore, incorporating multiple independent mutations reduces the likelihood that vaccine strains revert to wild type and become pathogenic. Several *S*. Typhi vaccine candidates have advanced to Phase 1 clinical trials and one such candidate is M01ZH09, a *S*. Typhi Ty2 Δ*aroC* Δ*ssaV* mutant, which has limited intracellular replication due to the *ssaV* deletion in the Type 3 Secretion System (T3SS) and the *aroC* mutation which results in a requirement for aromatic amino acids for growth (Kirkpatrick *et al*. 2006). This vaccine did not cause bacteremia, fever or fecal shedding for an extended duration in volunteers but was able to induce antibody responses to lipopolysaccharide (LPS) and flagellin; however, when tested in a human challenge model, a single dose of M01ZH09 did not protect volunteers from developing typhoid fever when challenged with a virulent strain of *S*. Typhi (Darton *et al*. 2016). The Center for Vaccine Development and Global Health (CVD) has also produced several typhoid vaccine candidates including a series of *S*. Typhi Ty2 mutants that are able to elicit robust humoral and cellular immune responses (Tacket and Levine 2007). The leading candidate, CVD 909, has mutations in *aroC* and *htrA* and has been engineered to constitutively express Vi polysaccharide (Wang *et al*. 2000). CVD 909 elicited Vi‐specific immune responses in addition to anti‐LPS and anti‐flagellin responses after a single dose making it an ideal typhoid vaccine candidate (Tacket *et al*. 2004).

While many NTS vaccine candidates have been developed and preclinically assessed, few have been tested in clinical trials. At the CVD, we have developed *S*. Typhimurium, *S. *Enteritidis and *S. *Newport vaccines with *guaBA* (encodes guanine biosynthesis), *clpPX* (encodes a regulatory protease), *htrA* (encodes heat‐shock protein) and *pipA* (part of *Salmonella* pathogenicity island [SPI] 5 which contributes to fluid accumulation in the intestine) deletions (Tennant *et al*. 2015; Higginson *et al*. 2018; Fuche *et al*. 2019). Live attenuated NTS vaccines from other groups have included deletions in *aroA*, *ssaV*, *crp*, *cdt*, *phoPQ*, *purB*, dam, Lon protease and *hfq* among many others (Matsui *et al*. 2003; Allam *et al*. 2011; Tennant and Levine 2015; Galen *et al*. 2016). Candidates that have advanced to clinical trials include a *S. *Typhimurium LH1160 vaccine engineered with deletions in the virulence genes *phoP* and *phoQ* and *purB* to eliminate purine biosynthesis and which expressed *Helicobacter pylori* urease from a plasmid. Volunteers receiving a single dose of this vaccine‐generated IgA and IgG antibody responses to *Salmonella* LPS and flagellin as well as detectable responses to *H*. *pylori* urease (Angelakopoulos and Hohmann 2000) indicating that attenuated NTS vaccines can be used to immunize against heterologous antigens. Another candidate that advanced to a Phase 1 clinical trial combined mutations in *aroA* and *ssaV* in an early Typhimurium version of the Typhi vaccine strain M01ZH09 discussed above. This vaccine, designated WT05, was well‐tolerated and elicited immune responses in most volunteers but was shed for 23 days (Hindle *et al*. 2002) which raises concerns about possible transmission to vulnerable populations. Below, we describe new developments to improve the safety and immunogenicity of live attenuated *Salmonella* vaccines with an emphasis on strategies to enhance acid resistance and reduce the potential for transmission.

### Live attenuated vaccines enhanced for acid resistance

To induce a robust and strongly efficacious immune response, vaccine organisms delivered orally must successfully transit the gastric acid barrier, passing into the intestinal tract in sufficient numbers to reach immune inductive sites and elicit both innate and adaptive responses. It has been hypothesized that passage through the gastric acid barrier induces critical virulence factors in *Salmonella* that prepare vaccine organisms for the eventual invasion and persistence within immune tissues (Galen *et al*. 2016). Historically, oral administration of live attenuated *Salmonella* vaccines has been carried out after administration of bicarbonate to fasting subjects to neutralize stomach acidity; an alternate method involves oral administration of vaccine contained within enteric coated acid resistant capsules which transit the stomach and subsequently deliver vaccine organisms directly into the intestinal tract. However, it has been postulated that bypassing the gastric acid barrier may also abort induction of SPI 1 and 2, potentially reducing the number of vaccine organisms able to invade intestinal tissues and trigger immunity (Brenneman *et al*. 2013, 2014). Several groups have therefore re‐engineered candidate vaccine strains such that acid resistance can be induced on demand in vitro during vaccine preparation under routine growth conditions such that organisms are immediately prepared for exposure to acid prior to oral administration, and more organisms can successfully pass through the gastrointestinal tract to reach immune inductive sites (Brenneman *et al*. 2013, 2014; Dharmasena *et al*. 2016a). Two approaches have been used to enhance the acid resistance of live attenuated *Salmonella* candidate vaccines. The first strategy involves re‐engineering endogenous extreme acid resistance pathways to enable induction of resistance on demand during vaccine production (Brenneman *et al*. 2013, 2014); the second method introduces exogenous acid resistance systems from other more acid‐resistant enteric pathogens such as *Shigella* (Dharmasena *et al*. 2016a).

**Figure 1:**
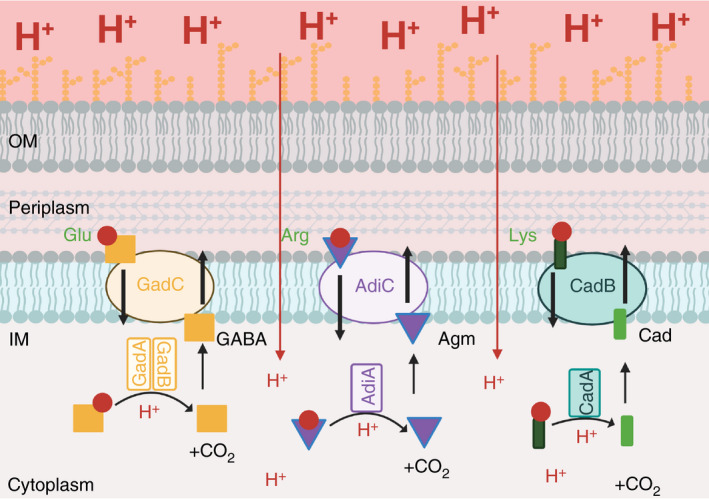
Endogenous and exogenous acid resistance pathways exploited in *Salmonella* vaccines. The diagram illustrates the two‐component amino acid decarboxylase‐antiporter resistance (AR) systems that have been utilized in candidate *Salmonella* vaccines to increase acid resistance. These systems are from left to right—the glutamic acid–dependent acid resistance system (GDAR, yellow) comprised of the GadC glutamate/γ‐aminobutyric acid (GABA) antiporter and glutamate decarboxylases, GadA/GadB; the arginine‐dependent acid resistance system (ADAR, purple) comprised of the AdiC arginine/agmatine antiporter (Agm) and the inducible arginine decarboxylase, AdiA; and the lysine‐dependent acid resistance system (LDAR, green) comprised of the CadB lysine/cadaverine antiporter (Cad) and the cytoplasmic inducible lysine decarboxylase, CadA. The ADAR and LDAR systems are native to *Salmonella*, while the GDAR system has been imported from *Shigella* into *Salmonella* strains to further enhance acid resistance. In low pH conditions (deep pink colour extracellular to the bacteria, large H^+^) and in the presence of their cognate amino acid, the decarboxylase raises intracellular pH (indicated by the light‐coloured cytoplasm) by decarboxylating the amino acid substrate (indicated by the orange square, purple triangle and green rectangle) in a proton‐dependent manner releasing CO_2_ in the process. The product binds the substrate‐specific antiporter in the inner membrane and exchanges the decarboxylated substrate with a new amino acid. IM, inner membrane; OM, outer membrane. Figure created with BioRender.com.

*Salmonella* possess at least three acid resistance pathways, all of which rely on a two‐component decarboxylase–antiporter system that acts upon cognate‐specific amino acid substrates as shown in Fig. 1 (Zhao and Houry 2010). These pathways increase acid survival of bacteria under extreme acidic conditions as low as pH 2·5. Acid resistance systems are typically induced under low pH conditions in the presence of the specific amino acid required for decarboxylation; induction is also controlled by anaerobiosis. Upon induction under acid stress conditions, the decarboxylase raises intracellular pH by consuming one cytoplasmic proton per decarboxylation reaction to liberate CO_2_. The product of this enzymatic reaction is then transported out of the cytoplasm through binding to an inner membrane substrate‐specific antiporter that exchanges the modified substrate with a new amino acid to continue the cycle (Zhao and Houry 2010).

The endogenous *Salmonella* arginine decarboxylase acid resistance system was re‐engineered in a candidate typhoid vaccine strain of *S*. Typhi by Brenneman *et al*. (2013), such that acid resistance became inducible on demand in the presence of the sugar rhamnose and the amino acid arginine. To accomplish this, the *adiA* gene encoding arginine decarboxylase was genetically arranged in tandem with the *adiC* gene encoding the arginine–agmatine antiporter AdiC and the resulting cassette placed under the transcriptional control of a rhamnose‐inducible rha‐P_BAD_ promoter cassette. The resulting vaccine organisms displayed significantly improved survival in vitro at pHs of 3·0 and 2·5. However, resistance levels did not exceed those conferred by the unmodified native decarboxylase system when organisms were induced under the more restrictive anaerobic growth conditions (Brenneman *et al*. 2013). To test the acid resistance of these organisms in vivo, strains were administered orally using a low gastric pH histamine mouse model (Brenneman *et al*. 2014) and surviving organisms enumerated from murine gastrointestinal tissue 1‐h post‐inoculation; acid resistance was assessed using a competition assay in which acid‐sensitive unmodified *S*. Typhi candidate vaccines were mixed 1 : 1 with acid‐resistant isogenic *S*. Typhi strains and competitive indices quantitated post inoculation. In vivo survival of *S*. Typhi acid‐resistant strains increased by ˜ 10‐fold when carrying the inducible arginine decarboxylase system (Brenneman *et al*. 2014).

Introduction of exogenous acid resistance systems has resulted in more robust improvement of acid resistance in candidate *Salmonella* vaccines. Dharmasena *et al*. (2016a) integrated a cassette encoding the glutamine decarboxylase system from *Shigella flexneri* 2a into the chromosome of the licensed *S*. Typhi typhoid vaccine Ty21a. This strategy was based on the observation that *Shigella* is substantially more resistant to acid stress than *Salmonella*, possibly due to the presence of a glutamine decarboxylase resistance pathway, not present in *Salmonella*, that confers the strongest resistance to extreme acid stress conditions (Zhao and Houry 2010). Therefore, a synthetic operon was constructed encoding the two paralogous GadA and GadB decarboxylases and the inner membrane antiporter GadC under the transcriptional control of the arabinose‐inducible AraC‐P_BAD_ promoter; expression was tested for low copy number plasmid‐based expression of this cassette versus expression when stably integrated into the chromosome. As expected, when acid survival was tested in vitro, plasmid‐based acid resistance increased by 5 log_10_ versus unmodified Ty21a after 3 h at pH 2·5 (Dharmasena *et al*. 2016a); when Ty21a carrying a chromosomally integrated glutamine acid resistance system was exposed to pH 2·5 for 3 h, survival was still increased by 4 log_10_ although survival depended on pre‐induction with cultures grown at pH 5·5 for 24 h prior to acid challenge (Dharmasena *et al*. 2016a).

### Optimization of vaccines for reduced fecal shedding and transmissibility

In the development of live attenuated oral vaccines, significant consideration must be given to limiting the length of time that vaccine strains are shed into the feces. Long periods of shedding into the environment can increase the opportunities for transfer of genetic elements or transmission of vaccine strains to unvaccinated, immunocompromised individuals (Davison 2002; Anderson 2008). Balancing tolerability with immunogenicity can be challenging as overattenuation may result in strains with limited shedding but that transit the gut so quickly that they fail to elicit protective immune responses. Among the approaches that have been advanced to develop live attenuated, *Salmonella* strains with limited fecal shedding and hence reduced transmissibility to unintended targets are gene mutations that limit colonization and survival, and strains engineered to self‐destruct after several rounds of replication.

Persistence in the intestine has been attributed to many factors including adhesins required for attachment and colonization. However, few genes have been directly implicated in fecal shedding (Gal‐Mor 2019). Two factors, *shdA* and *ratB*, were identified on a genetic island unique to serovars within *S*. *enterica* subspecies I, which infect avian and mammalian species, but absent in subspecies II‐VII (Kingsley *et al*. 2000, 2003). *ShdA* encodes a fibronectin‐binding protein belonging to the autotransporter Type 5 secretion system (T5SS) (Kingsley *et al*. 2004); an *S*. Typhimurium strain with *shdA* and *aroA* mutations was shed in the feces of BALB/c mice intermittently and overall for a shorter time compared to a control *aroA* mutant strain in a reduced virulence model of infection (Kingsley *et al*. 2000, 2002). Directly upstream of *shdA* is *ratB* and in similar experiments Δ*ratB* mutants were shed for a shorter period from stool of BALB/c mice and CBA/J mice which are more resistant to lethal *S*. Typhimurium infection. In contrast, deletion of the *sivH* gene from this island did not limit shedding (Kingsley *et al*. 2003). An additional factor contributing to fecal shedding is the *misL* gene which, like *shdA*, encodes a T5SS protein that binds fibronectin (Dorsey *et al*. 2005). Unlike *shdA* and *ratB*, *misL* is located on SPI3. A *misL* mutant, MCL1, was not deficient in colonization but was recovered from cecum, spleen and mesenteric lymph nodes at much lower numbers than a competing *phoN* mutant strain which was not deficient in colonization. Further, a *shdA misL* double mutant, CWD9, was recovered in lower amounts than a *shdA* mutant in competition experiments; however, this difference was not statistically significant (Dorsey *et al*. 2005). In parallel development towards a live vaccine with reduced shedding, *shdA* and *misL* mutations were introduced into candidate *S*. Typhimurium vaccine SL3261 (an *aroA* mutant), either singly or combined. Significantly fewer bacteria were detected in feces of mice 14 days after oral delivery of either the single or double mutant compared to a control strain (Abd El Ghany *et al*. 2007). Additionally, introduction of these mutations into a vaccine strain carrying a plasmid for expression of a heterologous antigen, tetanus toxin fragment C (TetC), did not diminish or alter systemic IgA and IgG responses or seem to negatively impact cytokine production while also stimulating anti‐TetC antibody production. Most interestingly, a single dose of vaccine carrying either TetC alone or TetC in the presence of *shdA* and *misL* mutations was able to protect 90% and 100% of mice, respectively, from lethal *S*. Typhimurium challenge (Abd El Ghany *et al*. 2007).

### Reducing transmissibility by programmed attenuation and delayed lysis

Programmed bacterial cell lysis or delayed lysis is a methodology that ensures death of a candidate vaccine strain after colonization of immune tissues. This novel system was developed by Roy Curtiss III's group to reduce the shedding of recombinant‐attenuated *Salmonella* vaccine (RASV) strains that have been exquisitely engineered to deliver homologous and heterologous antigens to immune system tissues (Kong *et al*. 2008). The system tightly controls expression of enzymes required for synthesis of two components of the peptidoglycan layer of the cell wall, diaminopimelic acid (DAP) and muramic acid. Synthesis of DAP is controlled by aspartate semi‐aldehyde dehydrogenase (encoded by *asd*), and UDP‐*N*‐acetylglucosamine enolpyruvyl transferase (encoded by *murA*) is the first enzyme involved in muramic acid synthesis; placing both of the genes encoding these two enzymes under the transcriptional control of the arabinose inducible AraC‐P_BAD_ promoter ensures that in the absence of exogenously supplied arabinose vaccine strains engineered using this approach lyse due to the inability to synthesize the cell wall. Additional mutations introduced into this strain to promote bacterial lysis include deletion of genes encoding enzymes for GDP‐fucose synthesis (*Δ(gmd‐fcl)‐26*) which prevents synthesis of colonic acid which can rescue *asd* mutants and a mutation in *relA* (*ΔrelA1123*) which renders the bacteria incapable of responding to amino acid starvation and ensures death in vivo (Torok and Kari 1980; Whitfield 2006; Kong *et al*. 2008). These RASV strains exhibit no lethality in mouse models when delivered at considerably high doses and disseminate to and colonize lymphoid tissues. In the absence of arabinose during in vivo infection, these strains are engineered to lyse after several rounds of replication and are eventually cleared from the liver, spleen and Peyer's patches. Moreover, they have been shown to elicit serum IgG and mucosal IgA antibodies and stimulate antigen‐specific cytokine production from T‐cells.

### *Salmonella* live carrier vaccines

The use of live attenuated bacterial vaccines as carriers for mucosal delivery of foreign antigens to stimulate the mucosal immune system was first proposed over three decades ago. This novel strategy aimed to induce immunity against at least two distinct pathogens using a single bivalent carrier vaccine. It was first tested using a live attenuated *S. *Typhi Ty21a strain (5076‐1C) in clinical trials in 1984 (Tramont *et al*. 1984), with modest but promising humoral immune responses elicited against the foreign antigen. However, this candidate live attenuated vaccine strain proved to be genetically unstable, and promising initial clinical results could not be consistently repeated in later studies (Black *et al*. 1987; Herrington *et al*. 1990). Since then, clinical trials with additional *Salmonella*‐based carrier vaccines have been conducted, with most vaccines derived from attenuated *S*. Typhi. As with the original trial, only modest foreign antigen‐specific immunity has been achieved in most cases, despite incorporation of incremental improvements in antigen expression technologies and carrier design over the years.

Several critically important factors influence the quality of an adaptive immune response against carrier vaccines and against the foreign antigen(s) they present to the immune system. Based on data from both murine intranasal animal models (Galen *et al*. 1997; Pasetti *et al*. 2000; Pickett *et al*. 2000) and clinical trials (Bumann *et al*. 2001; Metzger *et al*. 2004; Khan *et al*. 2007; Bumann *et al*. 2010), several key factors influence carrier‐ and foreign antigen‐specific immunity, including carrier strain genetics, antigen expression strategies, immunization protocols and human host factors (Galen and Curtiss 2014; Clark‐Curtiss and Curtiss 2018). Clearly, several different strategies can be either engineered or carefully chosen to minimize any detrimental influence that each of these factors may exert on carrier vaccine immunogenicity. Here, we will focus our discussion on key concepts relevant to recent advances in *Salmonella‐*based carrier vaccine development.

Since the first clinical trials conducted with carrier vaccines in the 1980s (Tramont *et al*. 1984; Black *et al*. 1987; Herrington *et al*. 1990), significant efforts have been invested in expression technologies for optimizing production of foreign antigens to stimulate biologically relevant immunity including either mucosal, humoral or cellular immune responses (Roland and Brenneman 2013; Galen and Curtiss 2014; Clark‐Curtiss and Curtiss 2018). It is well appreciated that carrier vaccines derived from attenuated *S*. Typhi are fully capable of eliciting mucosal, humoral and cellular responses after oral immunization in humans (Levine *et al*. 2001; Sztein 2007; Booth *et al*. 2017). However, the design of carrier vaccines presents unique challenges posed directly by expression of the foreign antigen that are not encountered in the engineering of attenuated parental vaccines per se. In addition to the potential toxicity of a given foreign antigen when expressed in a carrier vaccine, which can directly interfere with the viability of the strain, inappropriate expression of high levels of an otherwise tolerable foreign antigen can stress the metabolic fitness of a carrier vaccine (Boe *et al*. 1987; Bailey 1993; Glick 1995; Zahn 1996; Corchero and Villaverde 1998). We and others have written extensively on this topic and will not recapitulate detailed arguments herein but rather refer the reader to more extensive discussions of mechanisms published elsewhere (Roland and Brenneman 2013; Galen and Curtiss 2014; Clark‐Curtiss and Curtiss 2018). Here, we emphasize that inappropriately high expression levels of foreign antigens create a physiological burden on a vaccine strain. This reduction in fitness is functionally equivalent to overattenuation of the vaccine strain, reducing both the replication of these organisms and their ability to reach immune inductive sites at sufficient levels to elicit both innate and adaptive immunity.

### Expression of foreign antigens from the chromosome

Overattenuation of carrier vaccines due to the metabolic burden associated with inappropriate expression of foreign antigens often occurs when these antigens are expressed from plasmids of high copy number. Plasmid‐based expression of foreign antigens exerts metabolic pressure on the carrier vaccine that can induce the spontaneous loss of the plasmid in vivo to improve growth rate (Galen and Curtiss 2014). This point is clearly illustrated by the early efforts by Osorio *et al*. (2009) to develop a live oral Ty21a‐based vaccine against anthrax focused on targeting the 83‐kDa full‐length Protective Antigen (PA83) of anthrax toxin, expressed from a multicopy expression plasmid. Placing the gene encoding PA83 under the transcriptional control of a strong constitutive P*_lpp_* promoter induced loss of the plasmid in 98% of passaged colonies after 50 generations of propagation in the antibiotic selection for the plasmid. Combining transcriptional control using an environmentally regulated P*_htrA_* promoter with an HlyA‐derived antigen export system significantly improved plasmid stability and antigen expression, eliciting high levels of PA83‐specific serum IgG responses and toxin neutralizing antibody (TNA) titres in immunized mice and 100% protection against a lethal aerosol challenge with 20 times the 50% lethal dose (LD_50_) of purified anthrax spores (Osorio *et al*. 2009).

After considering both the risk of plasmid instability in vivo, as well as the need for plasmid selection using antibiotics (currently discouraged by the US Food and Drug Administration for live vaccines intended for human oral immunization), the candidate Ty21a‐derived anthrax vaccine described above was re‐engineered to express PA83 from a genetic cassette integrated into the chromosome (Sim *et al*. 2017). To accommodate the loss of copy number after removal from plasmids, the integrated cassette was placed under the transcriptional control of the strong constitutive P*_lpp_* promoter; however, subsequent passaging of the resulting candidate vaccine strain resulted in 100% genetic stability and excellent expression of PA83 after >100 generations of propagation. Despite a significant drop in TNA titres elicited in mice by chromosomally encoded antigen (9942, Sim *et al*. 2017) versus plasmid encoded antigen (16241, Osorio *et al*. 2009), 100% protection was observed after aerosol challenge with 5 LD_50_s of purified spores; interestingly 70% protection was also observed in vaccinated rabbits when aerosol challenged with 200 LD_50_s of purified fully virulent Ames spores. These data clearly demonstrate the feasibility of overcoming many of the potential toxicity, genetic stability and antibiotic resistance problems associated with plasmid‐based expression of foreign antigens in live carrier strains while still maintaining robust immunogenicity and protective efficacy against challenge.

The pace of engineering chromosomal expression of foreign antigens in Ty21a typhoid carrier vaccines has significantly increased in the past 5 years with efforts to develop bivalent vaccines against both typhoid fever and shigellosis caused by multiple serotypes of *Shigella*. These efforts have focused on the expression of protective *Shigella* O polysaccharide antigens on the surface of Ty21a. Since the operons encoding these antigens are typically >10 kilobases (kb) in length, it would be unlikely that such large cassettes would be genetically stable on multicopy expression plasmids. Therefore, chromosomal expression of such large foreign antigen cassettes was hypothesized to ensure stable expression that would potentially engender protection. This hypothesis was confirmed by Dharmasena *et al*. (2013) in a study that compared chromosomal versus plasmid‐based expression and immunogenicity of a large synthetic operon encoding the form I OPS of *Shigella sonnei* in Ty21a. When an ˜12‐kb genetic cassette encoding the *S*. *sonnei* O‐antigen biosynthetic operon was inserted into a low copy expression plasmid and introduced into Ty21a, expression of the heterologous O antigen was observed in Ty21a but attempts to remove the antibiotic resistance marker to improve clinical acceptability resulted in significant plasmid instability. After integration into the chromosome, excellent expression of form I O‐antigen was observed, and 100% genetic stability was demonstrated over 75 generations of propagation. Robust titres of serum IgG against *S*. *sonnei* were observed after two intraperitoneal immunizations, and 100% protection against an intraperitoneal challenge with 100 LD_50_s of fully virulent *S*. *sonnei* was also observed (Dharmasena *et al*. 2013). These observations support the early observations documented in clinical trials with Ty21a strain 5076‐1C in which a chromosomally integrated cassette encoding *S*. *sonnei* engendered antigen‐specific serum immunity in volunteers and showed protection against severe dysentery in human challenge studies, despite the genetic instability of the construct (Black *et al*. 1987). Dharmasena *et al*. (2016b) have gone on to construct and test similar Ty21a bivalent vaccines stably expressing heterologous O antigens from *S*. *dysenteriae*, *S*. *flexneri* 2a and *S*. *flexneri* 3a (Dharmasena *et al*. 2017). Interestingly, attempts to elevate expression levels of the foreign O antigen by placing the operons under the transcriptional control of the strong constitutive P*_lpp_* promoter were not required to confer 100% protection against homologous challenge with *Shigella*, despite significantly enhancing O antigen expression versus native *Shigella* promoters.

Bivalent Ty21a vaccines against shigellosis have also been constructed by incorporating chromosomally encoded acid resistance operons and assessed by Wu *et al*. (2017) for immunogenicity and protective efficacy in mouse models. In contrast to studies mentioned above, in which mice were both immunized and challenged by the intraperitoneal route, mice in these experiments were immunized and challenged intranasally to more closely approximate the oral route of immunization intended for human use. This group successfully integrated both the acid resistance and *S*. *sonnei* O antigen operons tandemly into the same chromosomal locus employed by previous investigators (the inactive Vi locus of Ty21a). This tandem set of chromosomal insertions resulted in robust expression of acid resistance, cell surface expression of the heterologous O antigen, and high levels of LPS‐specific serum IgG responses. Most importantly, protective efficacy was 55% for mice immunized with an isogenic strain without the acid resistance cassette, but efficacy improved to 85% for mice receiving isogenic vaccines encoding for acid resistance (Wu *et al*. 2017). These results suggest that a single chromosomal locus can be effectively exploited for the simultaneous expression of immunogenic levels of multiple vaccine antigens that could not otherwise be stably expressed either on a single expression plasmid or multiple expression plasmids contained within a single carrier strain.

### Exploiting *Salmonella* secretion systems in live carrier vaccines

The T3SS of *Salmonella* provides an additional mechanism for the development of live vector vaccines against *Salmonella* and other pathogens. T3SS translocate effector proteins directly to the host cell cytoplasm and SPI1 and SPI2 translocate effectors at different phases of infection. These systems in *Salmonella* are among the best studied systems in Gram‐negative bacteria, and their composition and functions have been thoroughly reviewed (Pinaud *et al*. 2018; De Souza Santos and Orth 2019; Miletic *et al*. 2020).

Fusion of heterologous antigens to T3SS effectors has been a successful strategy for development of *Salmonella* as live vector vaccines and fusion to various effector proteins have mediated export to the endosomal compartment and elicited protective immune responses. This is particularly advantageous for immunization against non‐*Salmonella* diseases. Using Typhimurium strain 12023 with mutations in a variety of genes including *htrA*, *purD*, *galE* or *htrA/purD* as carrier strains, fragments of *Listeria monocytogenes* listeriolysin (*lisA*, Llo) and p60 (*iap*) were fused to the N‐terminal translocation signal of the effector SseF (Husseiny *et al*. 2007). Translocation of fusion proteins was detected in bone‐marrow derived dendritic cells and a macrophage‐like cell line. Importantly, mice immunized with these constructs had reduced organ burden 8 weeks after challenge with a sublethal dose of *L*. *monocytogenes* (Husseiny *et al*. 2007). Further development of this system demonstrated that fusion of ovalbumin (OVA) and listeriolysin to the effector SseJ stimulated OVA‐specific T‐cells in vitro and Llo‐specific cytotoxic T‐cells which are important for clearance of *L*. *monocytogenes* (Hegazy *et al*. 2012).

This strategy has also been applied to development of an anti‐cancer vaccine. The tumour‐associated antigen, survivin, optimally expressed as a fusion protein with SseJ was translocated to the cytoplasm of bone marrow‐derived dendritic cells demonstrating efficient export of a tumour antigen. Oral delivery of this vaccine strain resulted in CD8 T‐cell infiltration and shrinking of tumours (Xu *et al*. 2014). This engineering has been adapted to CVD 908‐*htrA*, and this vaccine candidate will be assessed in a Phase 1 clinical trial for treatment of multiple myeloma (ClinicalTrials.gov Identifier: NCT03762291). Most recently, fusion of *Pseudomonas aeruginosa* V antigen (PcrV) to SseJ was successfully exported into RAW264.7 macrophage‐like cells when expressed by Typhimurium strain 14028 (Aguilera‐Herce *et al*. 2019). The PcrV‐SseJ fusion protein was exported from a Typhimurium vaccine strain SV9699 (*aroA551*::*Tn10 ΔaroB*::Km) and induced high titres of PcrV‐specific IgG in mice after a single intraperitoneal dose. Mice subsequently challenged with a lethal dose of *P*. *aeruginosa* PAO1 had reduced bacterial loads in the lung and spleen and were significantly protected from challenge in comparison to immunization with vector only (Aguilera‐Herce *et al*. 2019).

One of the few live *Salmonella* vaccines expressing a foreign antigen to be tested in clinical trials includes an HIV‐1 vaccine developed by engineering a plasmid to encode the first 104 amino acids of SopE fused to a monoclonal antibody tag (M45), Influenza A nucleoprotein (NP), and a mutant HIV Gag protein codon optimized for T3SS. This plasmid was introduced into *S*. Typhimurium strain SL1344 carrying multiple mutations, *ΔphoP/ΔphoQ aroA*, *strA/strB* and *asd* to produce vaccine strain CKS257 (Kotton *et al*. 2006). Preclinical studies using a simian immunodeficiency virus (SIV)‐specific construct demonstrated Gag epitope‐specific CD4^+^ and CD8^+^ T‐lymphocytes in immunized rhesus macaques but animals were not protected from challenge with SIV (Evans *et al*. 2003). In a Phase 1 safety trial, volunteers developed *Salmonella‐*specific IgA and IgG titers but no antibodies against the HIV Gag protein suggesting that insufficient protein was expressed and/or translocated.

## Use of recombinant *Salmonella* as reagent strains for the production of antigens used in other vaccine formats

*Salmonella* strains engineered using the strategies discussed above have additional value in their capacity as sources of bacterial surface components for parenteral vaccines. Attenuation allows for them to be grown to high concentration for high yield of products with reduced risk to personnel. These bacterial components can subsequently be used as purified antigens for subunit or conjugate vaccines. Conjugate vaccines are composed of polysaccharides covalently linked to a protein, and while polysaccharides alone are poor immunogens, particularly in children, the addition of protein carriers boosts humoral and cellular immune responses (Rappuoli 2018). Typbar‐TCV, a vaccine composed of the Vi polysaccharide of *S*. Typhi conjugated to tetanus–toxoid, had an efficacy of over 80% in Phase 3 trials in children aged 9 months to 16 years in Nepal (Shakya *et al*. 2019). It remains to be seen how long this protection will last, but its efficacy in children makes it an important tool in reducing typhoidal *Salmonella* infections in LMICs.

NTS do not express capsular polysaccharides like Vi; however, OPS does elicit antibody responses that mediate clearance of bacteria (Baliban *et al*. 2017; Micoli *et al*. 2018; Schuster *et al*. 2019). The diversity in O‐antigens presents a challenge in developing a broadly protective vaccine but the ability to produce large quantities of specific O‐antigens and proteins from corresponding serogroups make the production of a multivalent glycoconjugate vaccine more feasible. Outer membrane vesicles (OMVs) represent a parallel strategy as the polysaccharide and protein components are already closely associated with the bacterial membrane. Below, we discuss progress in production of glycoconjugate and vesicular vaccines against NTS.

### *Salmonella* as a source of antigens for conjugate vaccines

The immune response to natural infection with *Salmonella* includes elevated antibody titres to the core and OPS (COPS). OPS alone is a poor immunogen and does not elicit helper T cell memory, but this can be overcome by chemical linkage to carrier proteins. Early studies using saccharides derived from phage‐mediated hydrolysis of an *S*. Typhimurium OPS demonstrated that mice were protected from homologous lethal challenge after active immunization with purified saccharides alone, saccharides conjugated to diphtheria toxin, tetanus toxin or outer membrane proteins (OMPs) (Jorbeck *et al*. 1979; Watson *et al*. 1992). Jorbeck *et al*. (1981) also raised antibodies in rabbits against similar vaccines and passively transferred that sera to mice prior to lethal challenge; transfer of these antibodies alone was sufficient to protect mice from infection suggesting that antibodies to OPS are important for limiting disease. In Phase 1 and 2 clinical trials, conjugates of *S*. Paratyphi A OPS linked to tetanus toxoid increased LPS‐specific IgG titers in volunteers, and these antibodies exhibited bactericidal activity that was abrogated by absorption with *S*. Paratyphi A LPS (Konadu *et al*. 2000).

The linkage of OPS to a variety of carrier proteins to produce glycoconjugate vaccines has been a major focus in the development of vaccines against *Salmonella*. While the protein partners in these glycoconjugates have standard sizes and share homology, OPS structure varies widely across and within species of Gram‐negative bacteria. Even within a single strain, multiple O antigen chain lengths are produced and vary depending on the growth phase. Controlling the chain length is important as the size of the polysaccharide can influence the immune response. Additionally, conjugate vaccines are highly defined and having reagent strains that produce O polysaccharide within a certain size range means that less effort needs to be expended to purify O polysaccharide of a specific molecular weight thereby enabling more economical production.

One of the major pathways controlling the synthesis of OPS in *Salmonella* is the Wzx/Wzy‐dependent pathway, a family of integral inner membrane proteins comprised of a Wzx flippase, Wzy polymerase and several other related Wzz polysaccharide chain‐length regulators (Islam and Lam 2014). The first step of the pathway involves a series of glycosyltransferase reactions which synthesize O‐antigen repeats on a lipid carrier, undecaprenyl pyrophosphate (UndPP). The Wzx flippase then translocates the UndPP‐linked O‐antigen across the inner membrane to the periplasmic side and transfers it to the Wzy polymerase for addition to a growing polymer. The Wzz regulator protein controls the number of OPS repeats in this polymer thereby controlling the length of LPS. *Salmonella* can encode more than one Wzz protein and each produces OPS of a different modal length. WzzB produces long‐chain OPS with lengths of 16–35 O‐antigen repeat units while the *Escherichia coli* Wzz homolog FepE, produces very long OPS with lengths of >100 O‐antigen repeating units (Murray *et al*. 2003).

Overexpression of *wzzB* cloned from *S*. Typhimurium I77, a clinical isolate from blood culture, resulted in increased expression of long relative to short and medium length OPS in *S*. Typhimurium CVD 1925, a reagent strain engineered for OPS purification for conjugate vaccines (Hegerle *et al*. 2018). Importantly, overexpression of *S*. Typhimurium *wzzB* in the candidate live attenuated vaccine strain *S*. Paratyphi A CVD 1902 also led to the production of OPS with chain lengths comparable to that of *S*. Typhimurium. Additionally, overexpression of a second *S*. Typhimurium *wzz* family member, *fepE*, produced very long OPS in heterologous systems.

**Figure 2:**
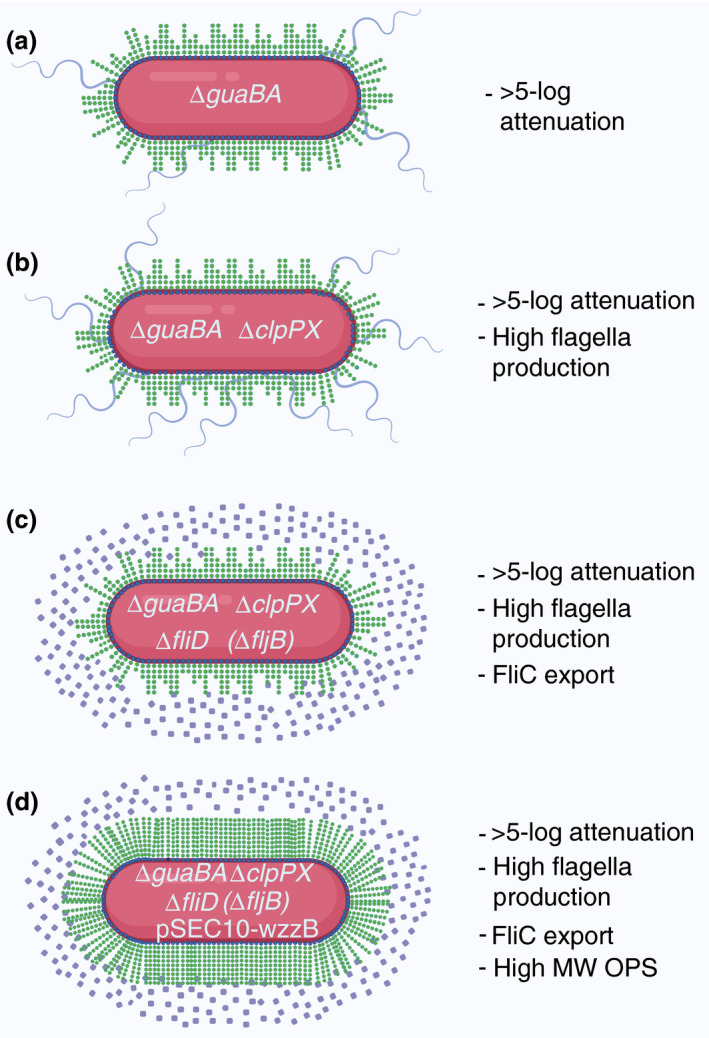
Reagent strains for the production of core and O‐polysaccharide (COPS) and flagellin. Live oral vaccine strains are highly attenuated and can be used to purify antigens safely and economically. This figure shows the deleted genes of reagent strains used for NTS COPS:FliC conjugate vaccines and the impact on phenotype and virulence particularly with respect to OPS (indicated by the green chains on the outer surface of the bacteria) length, flagellar (indicated as lavender hair‐like projections) expression, and flagellin protein FliC secretion (indicated as lavender circles). (a) Deletion of *guaBA* results in guanine auxotrophy and increases the LD_50_ by 5 log_10_. (b) The *clpP*X mutations results in hyperflagellated mutants. (c) Deletion of *fliD* eliminates the flagellar cap protein which results in monomers of the flagellin protein FliC being secreted into the media while deletion of *fljB* from *Salmonella* Typhimurium results in loss of phase 2 flagellin. (d) Expression of the protein that regulates the number of OPS repeats, *wzzB*, results in expression of long chain LPS and hence a more uniform product. Figure created with BioRender.com

At the CVD, we have developed conjugate vaccines against *S. *Typhimurium and *S. *Enteritidis which consist of COPS chemically linked to phase 1 flagellin proteins (FliC) from the homologous serovar (Baliban *et al*. 2017). We have constructed reagent strains to make purification of COPS and FliC safer, simpler, and more economical (Fig. 2). Deletion of *guaBA* and *clpP* (encodes a protease which regulates flagella expression) from *S*. Typhimurium I77 and *S*. Enteritidis R11 resulted in CVD 1921 and CVD 1941, respectively (Tennant *et al*. 2011). These strains require guanine for growth and are hyperflagellated and hyper motile. Subsequent deletion of *fliD* eliminates the cap protein of the flagellin and results in large quantities of monomeric FliC being released into the supernatant. The resulting strains CVD 1923 (*S*. Typhimurium Δ*guaBA* Δ*clpP* Δ*fliD*) and CVD 1943 (*S*. Enteritidis Δ*guaBA* Δ*clpP* Δ*fliD*) are highly attenuated and have an LD_50_ greater than 5 log_10_ above their parental strains (Tennant *et al*. 2011). Deletion of the gene that encodes Phase 2 flagella, FljB, in *S*. Typhimurium ensures that only Phase 1 flagellin is produced by our reagent strain, CVD 1925. Since *S. *Typhimurium produces mostly short length OPS, we overexpressed *wzzB* from a plasmid to ensure that medium to long chain OPS was produced consistently (Baliban *et al*. 2017; Hegerle *et al*. 2018). Importantly, because these strains are highly attenuated, they can be grown to high concentration by fermentation, and flagellin monomers can be purified directly from the culture supernatant (Simon *et al*. 2014) while OPS can be extracted from the bacterial cell mass. Conjugate vaccines produced using COPS and FliC from *S. *Typhimurium CVD 1925 (pSEC10‐*wzzB*) and *S. *Enteritidis CVD 1943 were immunogenic and able to protect mice against lethal challenge with wild‐type NTS (Simon *et al*. 2011; Baliban *et al*. 2017). These vaccines are currently being evaluated in a Phase 1 clinical trial (ClinicalTrials.gov Identifier: NCT03981952).

### Outer membrane vesicles

**Figure 3:**
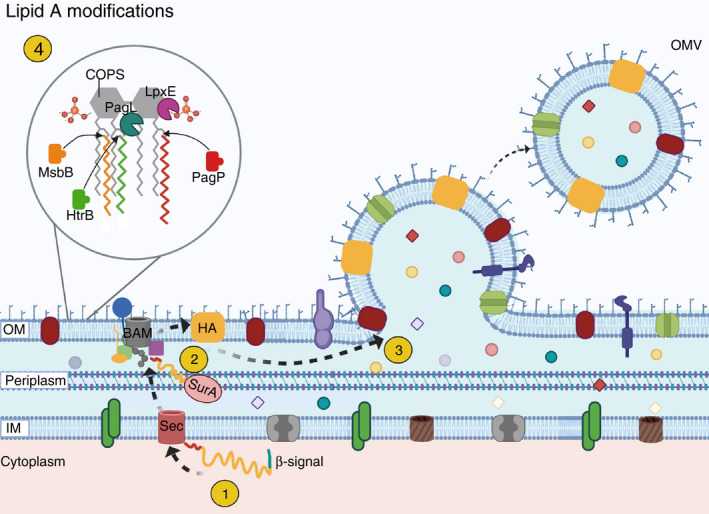
Production of outer membrane vesicles (OMVs) and lipid A modifications. OMVs are spherical vesicles enriched with proteins. Their production can be manipulated to express antigens from unrelated pathogens as shown in this figure. Black dashed arrows indicate the movement of the antigen from cytoplasm to outer membrane (OM). (1) An unfolded heterologous antigen (HA) in the periplasm (yellow line with an N‐terminal secretion signal in red and a C‐terminal β‐signal in blue) is translocated from the cytoplasm across the inner membrane (IM) via the Sec translocase and the chaperone protein SurA directs HA to the BAM complex. (2) The BAM complex facilitates proper folding of HA and its insertion into the OM. (3) Vesicles subsequently bud from the OM and contain OM proteins, LPS, periplasmic proteins, and the foreign antigen. (4) The inset shows lipid A modifications used to reduce the reactogenicity of OMVs. Black solid arrows indicate the position at which the acyl chain (zigzag lines) modifications are made. These include deletion of HtrB which blocks the addition of a 12‐carbon secondary chain to the 2′ position; deletion of MsbB which blocks the addition of a 14‐carbon secondary chain to the 3′ position; deletion of PagP which blocks the addition of a 16‐carbon secondary chain to the existing acyl chain at the 2 position. PagL is a deacylase that removes the β‐hydroxymyristoyl chain at the carbon‐3 position and LpxE dephosphorylates lipid A at the carbon 1 position. COPS, core and O polysaccharide; IM, inner membrane. Figure created with BioRender.com.

In addition to their use for the synthesis of conjugate vaccines, reagent strains derived from *Salmonella* have also recently been used for production of OMVs (Fig. 3). OMVs are small approximately spherical exosomes that are spontaneously shed from Gram‐negative bacteria. They range in size from ˜25 to 250 nm in diameter (approximately one tenth the size of the originating bacterium) and are comprised predominantly of the endogenous proteins, lipoproteins and LPS found in the bacterial outer membrane, in addition to other smaller amounts of peptidoglycan, periplasmic proteins and other cellular constituents (Schwechheimer and Kuehn 2015; van der Pol *et al*. 2015; Rossi *et al*. 2016; De Benedetto *et al*. 2017). Due to the robust immunogenicity of these vesicles and their relative ease of manufacture, significant interest in the use of OMVs as vaccines against a variety of human pathogens has rapidly expanded the genetic and pharmaceutical technologies involved in development of this novel vaccination strategy.

Purified OMVs from *S*. Typhimurium have been reported to be immunogenic facsimiles of wild‐type *S*. Typhimurium, capable of inducing activation and maturation of murine macrophages and dendritic cells in vitro, as well as antigen‐specific B cell and T cell CD4^+^ responses in vivo that resulted in protection against homologous challenge with virulent *S*. Typhimurium (Alaniz *et al*. 2007). Schetters *et al*. (2019) have also reported that purified *S*. Typhimurium OMVs are capable of antigen‐specific dendritic cell‐mediated cross‐presentation to CD8^+^ T cells. Interestingly, in these experiments, OMVs were engineered for expression of a heterologous ovalbumin model 98‐residue peptide. Such observations support the use of purified OMVs as an immunologically flexible vaccine modality, capable of inducing pathogen‐specific humoral and cellular immunity that potentially confers robust protection against disease.

Among the full array of biological molecules contained on OMVs are pathogen‐associated molecular patterns which act as powerful agonists to host innate pattern‐recognition receptors including toll‐like receptors (TLRs), responsible for triggering initial inflammatory responses to the presence of potential pathogens. The success of a candidate vaccine often rests on the balance between stimulating maximum protective immunity while at the same time minimizing clinically unacceptable inflammatory responses (i.e., systemic reactogenicity). For Gram‐negative bacteria, strong reactogenicity is often associated with the interaction of LPS with TLR4 complexed with myeloid differentiation factor 2 (TLR4/MD2); the principle LPS domain responsible for this interaction is the lipid A membrane anchoring portion of the molecule (Bertani and Ruiz 2018; Mancini *et al*. 2020). It has been demonstrated that using various combinations of naturally occurring bacterial acylating and deacylating enzymes involved in lipid A synthesis, it is possible to genetically engineer the structure of lipid A to generate a broad spectrum of immunostimulatory LPS variants ranging from fully reactogenic to non‐reactogenic entities (Needham *et al*. 2013).

Although production of OMVs from *Salmonella* and other Gram‐negative bacteria occurs naturally, release of these vesicles is strongly influenced by growth conditions and bacterial metabolism and is therefore not amenable to scale‐up for vaccine production. However, efficient production of OMVs from *Salmonella* can be easily facilitated by genetic deletion of the chromosomal *tolR* gene which disrupts the stable anchoring of the outer membrane to the inner membrane (Egan 2018). This strategy has been applied to the development of an OMV‐based vaccine against shigellosis derived from *S. sonnei* (1790GAHB); these Generalized Modules for Membrane Antigens (GMMAs) are highly immunogenic and have rapidly progressed through Phase 1 and 2 clinical trials (Launay *et al*. 2017, 2019; Obiero *et al*. 2017). The same strategy has also been applied to the development of a bivalent GMMA‐based vaccine against iNTS derived from invasive strains of both *S*. Typhimurium and *S*. Enteritidis, the two most prominent serovars in endemic areas of sub‐Saharan Africa (Gilchrist and MacLennan 2019).

GMMAs purified from *S*. Typhimurium and *S*. Enteritidis have been examined in detail for reactogenicity; interestingly, unmodified lipid A is present as a mixture of hepta‐acylated and hexa‐acylated species and as such was expected to be unacceptably reactogenic in monocyte activation test (MAT) assays. Therefore, gene deletions were introduced into parent strains targeting either PagP (catalyzes the addition of a 16 carbon secondary acyl chain to the existing acyl chain at position 2 of lipid A), MsbB (catalyzes the addition of a 14 carbon secondary chain to the 3′ position of lipid A), HtrB (catalyzes the addition of a 12 carbon secondary chain to the 2′ position of lipid A), or combinations of these deletions (Rossi *et al*. 2016). Construction of the modified *S*. Enteritidis strain Δ*tolR* Δ*msbB* Δ*pagP* resulted in purified OMVs containing LPS with uniformly penta‐acylated lipid A and a 30‐fold reduction in reactogenicity in the MAT assay (a level comparable to that of similarly engineered *S*. *sonnei* GMMAs tested in clinical trials), while the same engineered deletions in *S*. Typhimurium resulted in a penta‐acylated LPS with a 200‐fold reduction in reactogenicity (Rossi *et al*. 2016). Surprisingly, it was also reported that the engineering of less reactogenic lipid A can result in the shortening of O‐antigen repeats in the resulting LPS (De Benedetto *et al*. 2017). Although this effect was not observed for vesicles derived from *S*. Enteritidis, the effect was dramatic for engineered vesicles from *S*. Typhimurium, with the molar percent of O‐antigen to total LPS being reduced from 10% for Δ*tolR* vesicles to levels less than 1% for vesicles purified from Δ*tolR* Δ*msbB* and Δ*tolR* Δ*htrB* engineered strains; a triple Δ*tolR* Δ*msbB* Δ*pagP S*. Typhimurium mutant was not tested in these experiments. When used to immunize CD1 mice subcutaneously with two doses of adjuvanted *S*. Typhimurium OMVs (˜1 mg per dose) spaced 4 weeks apart, LPS‐specific serum IgG responses were comparable to unmodified vesicles from Δ*tolR* strains, but functional serum bactericidal titres dropped 3–5‐fold relative to unmodified vesicles (De Benedetto *et al*. 2017).

In an elegant study recently reported by Micoli *et al*. (2018), the immunogenicity and efficacy of unmodified OMVs from Δ*tolR* strains of *S*. Typhimurium and *S*. Enteritidis were compared to glycoconjugate vaccines in C57BL/6 mice immunized subcutaneously with two doses spaced 28 days apart and challenged intraperitoneally 17 days later with fully virulent *S*. Typhimurium or *S*. Enteritidis. Both OMV‐based and glycoconjugate vaccines were tested with or without the adjuvant Alhydrogel. OMVs were found to induce excellent O‐antigen‐specific humoral responses and serum bactericidal responses comparable to the conjugates, but without the use of adjuvant; unadjuvanted glycoconjugate vaccines were not immunogenic. However, the OMVs used in this study were not engineered for reduction in the reactogenicity of lipid A. Therefore, adsorption of OMVs to aluminium‐based adjuvants may still be necessary to improve the safety of these vaccines, based on the observation that the reactogenicity of LPS in purified OMVs can be reduced relative to free LPS by adsorption to aluminium‐based adjuvants (Rosenqvist *et al*. 1998). Such a solution may resolve the issue of reduced O‐antigen concentrations observed in OMVs purified from strains of *S*. Typhimurium engineered for reduction of lipid A reactogenicity.

### New directions for OMV‐based vaccines

The purified OMVs described above were all generated from Δ*tolR* mutants of *S*. Typhimurium and *S*. Enteritidis, further engineered by deletion of endogenous acylating enzymes to create less reactogenic vaccine candidates. However, it has been recently reported by Elhenawy *et al*. (2016) that overexpression (rather than deletion) of an endogenous lipid A modifying enzyme PagL in *S*. Typhimurium results in overproduction of OMVs by an unknown mechanism. PagL is a deacylase that removes the β‐hydroxymyristoyl chain at the carbon 3 position of lipid A. When overexpressed from a multicopy expression plasmid in *S*. Typhimurium, OMV production increased four fold (Elhenawy *et al*. 2016). This remarkable observation raises the intriguing possibility of easily introducing plasmids encoding PagL into other Gram‐negative strains to induce the formation of vesicles that could potentially be used as vaccines similar to the GMMA strategy.

Over the years we and others have developed attenuated strains of *S*. Typhi, several of which have been successfully tested in clinical trials (Galen *et al*. 2016). Our group has also developed genetically stabilized low copy number plasmids for expression of foreign antigens from unrelated human pathogens as candidate vaccines (Galen *et al*. 2010), as well as surface expression systems capable of moving such foreign antigens out to the surface of our attenuated vaccine strains (Galen *et al*. 2009, 2004). Expression of PagL in such attenuated strains raises the hypothesis of being able to produce recombinant OMVs (rOMVs) expressing foreign vaccine antigens on their surface in the context of TLR‐specific agonists capable of efficiently stimulating innate immunity and more robust adaptive responses. Through integration into the chromosome or further addition of other lipid A modifying enzymes such as LpxE from *Francisella tularensis* (Needham *et al*. 2013), which dephosphorylates lipid A at the carbon 1 position, it becomes theoretically possible to engineer *S*. Typhi candidate vaccine strains capable of producing naturally adjuvanted OMVs similar in reactogenicity profile to the clinically acceptable monophosphoryl lipid A (MPL) adjuvant. It has also been reported that vaccine antigen administered by two separate routes or formulations (i.e., a heterologous prime‐boosting strategy) results in more robust immunogenicity when compared to immunity elicited by either individual modality alone (Vindurampulle *et al*. 2004; Chinchilla *et al*. 2007; Ramirez *et al*. 2010). It may therefore be possible to use attenuated *S*. Typhi vaccine strains engineered for hyper‐expression of rOMVs in combination with purified rOMVs in a novel heterologous prime‐boost immunization regimen to elicit protective immunity against a wide variety of human pathogens.

## Conclusions

The global burden of *Salmonella* remains a concern given increasing antimicrobial resistance. However, the variety of *Salmonella* vaccine approaches under development is a promising sign of a future where this burden is minimized. Although live attenuated *Salmonella* vaccines are highly immunogenic and easy to administer, further research is needed to reduce fecal shedding of NTS vaccine strains to address regulatory concerns. Additional studies will be needed to determine whether live attenuated *Salmonella* vaccines will be safe and immunogenic in HIV‐infected or other immunocompromised individuals. Notably, *Salmonella* are also ideal vectors for the expression of heterologous antigens and can provide a flexible platform in the development of vaccines against other pathogens. Finally, the ease with which *Salmonella* can be manipulated has also allowed them to serve as reagent strains for safe and economical production of subunit, OMV‐based and conjugate vaccines.

## Conflict of Interest

Drs Galen and Tennant have multiple patents on *Salmonella* vaccines.

## References

[jam15055-bib-0001] Abd El Ghany, M. , Jansen, A. , Clare, S. , Hall, L. , Pickard, D. , Kingsley, R.A. and Dougan, G. (2007) Candidate live, attenuated *Salmonella enterica* serotype Typhimurium vaccines with reduced fecal shedding are immunogenic and effective oral vaccines. Infect Immun 75, 1835–1842. 17296764 10.1128/IAI.01655-06 PMC1865686

[jam15055-bib-0002] Aguilera‐Herce, J. , García‐Quintanilla, M. , Romero‐Flores, R. , McConnell, M.J. and Ramos‐Morales, F. (2019) A live *Salmonella* vaccine delivering PcrV through the type III secretion system protects against *Pseudomonas aeruginosa* . mSphere 4, e00116–00119. 30996108 10.1128/mSphere.00116-19 PMC6470209

[jam15055-bib-0003] Alaniz, R.C. , Deatherage, B.L. , Lara, J.C. and Cookson, B.T. (2007) Membrane vesicles are immunogenic facsimiles of *Salmonella* Typhimurium that potently activate dendritic cells, prime B and T cell responses, and stimulate protective immunity in vivo. J Immunol 179, 7692–7701. 18025215 10.4049/jimmunol.179.11.7692

[jam15055-bib-0004] Allam, U.S. , Krishna, M.G. , Lahiri, A. , Joy, O. and Chakravortty, D. (2011) *Salmonella enterica* serovar Typhimurium lacking *hfq* gene confers protective immunity against murine typhoid. PLoS One 6.e16667 10.1371/journal.pone.0016667 PMC3036662 21347426

[jam15055-bib-0005] Anderson, E.J. (2008) Rotavirus vaccines: viral shedding and risk of transmission. Lancet Infect Dis 8, 642–649. 18922486 10.1016/S1473-3099(08)70231-7

[jam15055-bib-0006] Angelakopoulos, H. and Hohmann, E.L. (2000) Pilot study of *phoP/phoQ*‐deleted *Salmonella enterica* serovar typhimurium expressing *Helicobacter pylori* urease in adult volunteers. Infect Immun 68, 2135–2141. 10722611 10.1128/iai.68.4.2135-2141.2000 PMC97395

[jam15055-bib-0007] Bailey, J.E. (1993) Host‐vector interactions in *Escherichia coli* . Adv Biochem Eng Biotechnol 48, 29–52. 8460576 10.1007/BFb0007195

[jam15055-bib-0008] Baliban, S.M. , Yang, M. , Ramachandran, G. , Curtis, B. , Shridhar, S. , Laufer, R.S. , Wang, J.Y. , Van Druff, J. *et al*. (2017) Development of a glycoconjugate vaccine to prevent invasive *Salmonella* Typhimurium infections in sub‐Saharan Africa. PLoS Negl Trop Dis 11, e0005493 10.1371/journal.pntd.0005493 PMC5397072 28388624

[jam15055-bib-0009] Bertani, B. and Ruiz, N. (2018) Function and biogenesis of lipopolysaccharides. EcoSal Plus 8, ESP‐0001‐2018. 10.1128/ecosalplus.esp-0001-2018 PMC6091223 30066669

[jam15055-bib-0010] Black, R.E. , Levine, M.M. , Clements, M.L. , Losonsky, G. , Herrington, D. , Berman, S. and Formal, S.B. (1987) Prevention of shigellosis by a *Salmonella* Typhi‐*Shigella sonnei* bivalent vaccine. J Infect Dis 155, 1260–1265. 2437220 10.1093/infdis/155.6.1260

[jam15055-bib-0011] Boe, L. , Gerdes, K. and Molin, S. (1987) Effects of genes exerting growth inhibition and plasmid stability on plasmid maintenance. J Bacteriol 169, 4646–4650. 3308847 10.1128/jb.169.10.4646-4650.1987 PMC213834

[jam15055-bib-0012] Booth, J.S. , Patil, S.A. , Ghazi, L. , Barnes, R. , Fraser, C.M. , Fasano, A. , Greenwald, B.D. and Sztein, M.B. (2017) Systemic and terminal ileum mucosal immunity elicited by oral immunization with the Ty21a typhoid vaccine in humans. Cell Mol Gastroenterol Hepatol 4, 419–437. 29022005 10.1016/j.jcmgh.2017.08.002 PMC5626924

[jam15055-bib-0013] Brenneman, K.E. , Willingham, C. , Kong, W. , Curtiss, R. III and Roland, K.L. (2013) Low‐pH rescue of acid‐sensitive *Salmonella enterica* serovar Typhi strains by a rhamnose‐regulated arginine decarboxylase system. J Bacteriol 195, 3062–3072. 23645603 10.1128/JB.00104-13 PMC3697538

[jam15055-bib-0014] Brenneman, K.E. , Willingham, C. , Kilbourne, J.A. , Curtiss, R. III and Roland, K.L. (2014) A low gastric pH mouse model to evaluate live attenuated bacterial vaccines. PLoS One 9.e87411 10.1371/journal.pone.0087411 PMC3906194 24489912

[jam15055-bib-0015] Bumann, D. , Behre, C. , Behre, K. , Herz, S. , Gewecke, B. , Gessner, J.E. , von Specht, B.U. and Baumann, U. (2010) Systemic, nasal and oral live vaccines against *Pseudomonas aeruginosa*: a clinical trial of immunogenicity in lower airways of human volunteers. Vaccine 28, 707–713. 19887136 10.1016/j.vaccine.2009.10.080

[jam15055-bib-0016] Bumann, D. , Metzger, W.G. , Mansouri, E. , Palme, O. , Wendland, M. , Hurwitz, R. , Haas, G. , Aebischer, T. *et al*. (2001) Safety and immunogenicity of live recombinant *Salmonella enterica* serovar Typhi Ty21a expressing urease A and B from *Helicobacter pylori* in human volunteers. Vaccine 20, 845–852. 11738748 10.1016/s0264-410x(01)00391-7

[jam15055-bib-0017] Chinchilla, M. , Pasetti, M.F. , Medina‐Moreno, S. , Wang, J.Y. , Gomez‐Duarte, O.G. , Stout, R. , Levine, M.M. and Galen, J.E. (2007) Enhanced immunity to *Plasmodium falciparum* circumsporozoite protein (PfCSP) by using *Salmonella enterica* serovar Typhi expressing PfCSP and a PfCSP‐encoding DNA vaccine in a heterologous prime‐boost strategy. Infect Immun 75, 3769–3779. 17502396 10.1128/IAI.00356-07 PMC1951980

[jam15055-bib-0018] Clark‐Curtiss, J.E. and Curtiss, R. III (2018) *Salmonella* vaccines: conduits for protective antigens. J Immunol 200, 39–48. 29255088 10.4049/jimmunol.1600608

[jam15055-bib-0019] Corchero, J.L. and Villaverde, A. (1998) Plasmid maintenance in *Escherichia coli* recombinant cultures is dramatically, steadily, and specifically influenced by features of the encoded proteins. Biotechnol Bioeng 58, 625–632. 10099300

[jam15055-bib-0020] Darton, T.C. , Jones, C. , Blohmke, C.J. , Waddington, C.S. , Zhou, L. , Peters, A. , Haworth, K. , Sie, R. *et al*. (2016) Using a human challenge model of infection to measure vaccine efficacy: a randomised, controlled trial comparing the typhoid vaccines M01ZH09 with placebo and Ty21a. PLoS Negl Trop Dis 10.e0004926 10.1371/journal.pntd.0004926 PMC4988630 27533046

[jam15055-bib-0021] Davison, J. (2002) Towards safer vectors for the field release of recombinant bacteria. Environ Biosafety Res 1, 9–18. 15612252 10.1051/ebr:2002001

[jam15055-bib-0022] De Benedetto, G. , Alfini, R. , Cescutti, P. , Caboni, M. , Lanzilao, L. , Necchi, F. , Saul, A. , MacLennan, C.A. *et al*. (2017) Characterization of O‐antigen delivered by generalized modules for membrane antigens (GMMA) vaccine candidates against nontyphoidal *Salmonella* . Vaccine 35, 419–426. 27998639 10.1016/j.vaccine.2016.11.089

[jam15055-bib-0023] De Souza Santos, M. and Orth, K. (2019) The role of the type III secretion system in the intracellular lifestyle of enteric pathogens. Microbiol Spectr 7, BAI‐0008‐2019. 10.1128/microbiolspec.bai-0008-2019 PMC11026088 31152523

[jam15055-bib-0024] Dharmasena, M.N. , Feuille, C.M. , Starke, C.E. , Bhagwat, A.A. , Stibitz, S. and Kopecko, D.J. (2016a) Development of an acid‐resistant *Salmonella* Typhi Ty21a attenuated vector for improved oral vaccine delivery. PLoS One 11.e0163511 10.1371/journal.pone.0163511 PMC5046385 27673328

[jam15055-bib-0025] Dharmasena, M.N. , Hanisch, B.W. , Wai, T.T. and Kopecko, D.J. (2013) Stable expression of *Shigella sonnei* form I O‐polysaccharide genes recombineered into the chromosome of live *Salmonella* oral vaccine vector Ty21a. Int J Med Microbiol 303, 105–113. 23474241 10.1016/j.ijmm.2013.01.001

[jam15055-bib-0026] Dharmasena, M.N. , Osorio, M. , Filipova, S. , Marsh, C. , Stibitz, S. and Kopecko, D.J. (2016b) Stable expression of *Shigella dysenteriae* serotype 1 O‐antigen genes integrated into the chromosome of live *Salmonella* oral vaccine vector Ty21a. Pathog Dis 74, ftw098. 27655911 10.1093/femspd/ftw098

[jam15055-bib-0027] Dharmasena, M.N. , Osorio, M. , Takeda, K. , Stibitz, S. and Kopecko, D.J. (2017) Stable chromosomal expression of *Shigella flexneri* 2a and 3a O‐antigens in the live *Salmonella* oral vaccine vector Ty21a. Clin Vaccine Immunol 24, e00181‐00117. 10.1128/CVI.00181-17 PMC5717188 29046309

[jam15055-bib-0028] Dorsey, C.W. , Laarakker, M.C. , Humphries, A.D. , Weening, E.H. and Baumler, A.J. (2005) *Salmonella enterica* serotype Typhimurium MisL is an intestinal colonization factor that binds fibronectin. Mol Microbiol 57, 196–211. 15948960 10.1111/j.1365-2958.2005.04666.x

[jam15055-bib-0029] Egan, A.J.F. (2018) Bacterial outer membrane constriction. Mol Microbiol 107, 676–687. 29315884 10.1111/mmi.13908

[jam15055-bib-0030] Elhenawy, W. , Bording‐Jorgensen, M. , Valguarnera, E. , Haurat, M.F. , Wine, E. and Feldman, M.F. (2016) LPS remodeling triggers formation of outer membrane vesicles in Salmonella. MBio 7, e00940–e1916. 27406567 10.1128/mBio.00940-16 PMC4958258

[jam15055-bib-0031] Evans, D.T. , Chen, L.M. , Gillis, J. , Lin, K.C. , Harty, B. , Mazzara, G.P. , Donis, R.O. , Mansfield, K.G. *et al*. (2003) Mucosal priming of simian immunodeficiency virus‐specific cytotoxic T‐lymphocyte responses in rhesus macaques by the *Salmonella* type III secretion antigen delivery system. J Virol 77, 2400–2409. 12551977 10.1128/JVI.77.4.2400-2409.2003 PMC141091

[jam15055-bib-0032] Feasey, N.A. , Dougan, G. , Kingsley, R.A. , Heyderman, R.S. and Gordon, M.A. (2012) Invasive non‐typhoidal *Salmonella* disease: an emerging and neglected tropical disease in Africa. Lancet 379, 2489–2499. 22587967 10.1016/S0140-6736(11)61752-2 PMC3402672

[jam15055-bib-0033] Fuche, F.J. , Jones, J.A. , Ramachandran, G. , Higginson, E.E. , Simon, R. and Tennant, S.M. (2019) Deletions in *guaBA* and *htrA* but not *clpX* or *rfaL* constitute a live‐attenuated vaccine strain of *Salmonella* Newport to protect against serogroup C2–C3 *Salmonella* in mice. Hum Vaccin Immunother 15, 1427–1435. 29927725 10.1080/21645515.2018.1491499 PMC6663134

[jam15055-bib-0034] Galen, J.E. , Buskirk, A.D. , Tennant, S.M. and Pasetti, M.F. (2016) Live attenuated human *Salmonella* vaccine candidates: tracking the pathogen in natural infection and stimulation of host immunity. EcoSal Plus 7, ESP‐0010‐2016. 10.1128/ecosalplus.esp-0010-2016 PMC5119766 27809955

[jam15055-bib-0035] Galen, J.E. , Chinchilla, M. , Pasetti, M.F. , Wang, J.Y. , Zhao, L. , Arciniega‐Martinez, I. , Silverman, D.J. and Levine, M.M. (2009) Mucosal immunization with attenuated *Salmonella enterica* serovar Typhi expressing protective antigen of anthrax toxin (PA83) primes monkeys for accelerated serum antibody responses to parenteral PA83 vaccine. J Infect Dis 199, 326–335. 19099487 10.1086/596066 PMC2626152

[jam15055-bib-0036] Galen, J.E. and Curtiss, R. III (2014) The delicate balance in genetically engineering live vaccines. Vaccine 32, 4376–4385. 24370705 10.1016/j.vaccine.2013.12.026 PMC4069233

[jam15055-bib-0037] Galen, J.E. , Gomez‐Duarte, O.G. , Losonsky, G.A. , Halpern, J.L. , Lauderbaugh, C.S. , Kaintuck, S. , Reymann, M.K. and Levine, M.M. (1997) A murine model of intranasal immunization to assess the immunogenicity of attenuated *Salmonella* Typhi live vector vaccines in stimulating serum antibody responses to expressed foreign antigens. Vaccine 15, 700–708. 9178472 10.1016/s0264-410x(96)00227-7

[jam15055-bib-0038] Galen, J.E. , Wang, J.Y. , Chinchilla, M. , Vindurampulle, C. , Vogel, J.E. , Levy, H. , Blackwelder, W.C. , Pasetti, M.F. *et al*. (2010) A new generation of stable, nonantibiotic, low‐copy‐number plasmids improves immune responses to foreign antigens in *Salmonella enterica* serovar Typhi live vectors. Infect Immun 78, 337–347. 19884333 10.1128/IAI.00916-09 PMC2798174

[jam15055-bib-0039] Galen, J.E. , Zhao, L. , Chinchilla, M. , Wang, J.Y. , Pasetti, M.F. , Green, J. and Levine, M.M. (2004) Adaptation of the endogenous *Salmonella enterica* serovar Typhi *clyA*‐encoded hemolysin for antigen export enhances the immunogenicity of anthrax protective antigen domain 4 expressed by the attenuated live‐vector vaccine strain CVD 908‐htrA. Infect Immun 72, 7096–7106. 15557633 10.1128/IAI.72.12.7096-7106.2004 PMC529119

[jam15055-bib-0040] Gal‐Mor, O. (2019) Persistent infection and long‐term carriage of typhoidal and nontyphoidal Salmonellae. Clin Microbiol Rev 32, e00088‐00018. 10.1128/CMR.00088-18 PMC6302356 30487167

[jam15055-bib-0041] Gayet, R. , Bioley, G. , Rochereau, N. , Paul, S. and Corthesy, B. (2017) Vaccination against *Salmonella* infection: the mucosal way. Microbiol Mol Biol Rev 81, e00007–e00017. 28615285 10.1128/MMBR.00007-17 PMC5584317

[jam15055-bib-0042] Germanier, R. and Fuer, E. (1975) Isolation and characterization of Gal E mutant Ty 21a of *Salmonella* Typhi: a candidate strain for a live, oral typhoid vaccine. J Infect Dis 131, 553–558. 1092768 10.1093/infdis/131.5.553

[jam15055-bib-0043] Gilchrist, J.J. and MacLennan, C.A. (2019) Invasive nontyphoidal *Salmonella* disease in Africa. EcoSal Plus 8, ESP‐0007‐2018. 10.1128/ecosalplus.esp-0007-2018 PMC11573285 30657108

[jam15055-bib-0044] Glick, B.R. (1995) Metabolic load and heterologous gene expression. Biotechnol Adv 13, 247–261. 14537822 10.1016/0734-9750(95)00004-a

[jam15055-bib-0045] Grimont, P.A.D. and Weill, F.‐X. (2007) Antigenic formulae of the Salmonella serovars: WHO Collaborating Centre for Reference and Research on Salmonella. Paris, France: Institut Pasteur Paris.

[jam15055-bib-0046] Hegazy, W.A.H. , Xu, X. , Metelitsa, L. and Hensel, M. (2012) Evaluation of *Salmonella enterica* type III secretion system effector proteins as carriers for heterologous vaccine antigens. Infect Immun 80, 1193–1202. 22252866 10.1128/IAI.06056-11 PMC3294654

[jam15055-bib-0047] Hegerle, N. , Bose, J. , Ramachandran, G. , Galen, J.E. , Levine, M.M. , Simon, R. and Tennant, S.M. (2018) Overexpression of O‐polysaccharide chain length regulators in Gram‐negative bacteria using the Wzx‐/Wzy‐dependent pathway enhances production of defined modal length O‐polysaccharide polymers for use as haptens in glycoconjugate vaccines. J Appl Microbiol 125, 575–585. 29603538 10.1111/jam.13772 PMC6726474

[jam15055-bib-0048] Herrington, D.A. , Van de Verg, L. , Formal, S.B. , Hale, T.L. , Tall, B.D. , Cryz, S.J. , Tramont, E.C. and Levine, M.M. (1990) Studies in volunteers to evaluate candidate *Shigella* vaccines: further experience with a bivalent *Salmonella* Typhi‐*Shigella sonnei* vaccine and protection conferred by previous *Shigella sonnei* disease. Vaccine 8, 353–357. 2204243 10.1016/0264-410x(90)90094-3

[jam15055-bib-0049] Higginson, E.E. , Ramachandran, G. , Panda, A. , Shipley, S.T. , Kriel, E.H. , DeTolla, L.J. , Lipsky, M. , Perkins, D.J. *et al*. (2018) Improved tolerability of a *Salmonella enterica* serovar Typhimurium live‐attenuated vaccine strain achieved by balancing inflammatory potential with immunogenicity. Infect Immun 86, e00440–e00418. 30249748 10.1128/IAI.00440-18 PMC6246900

[jam15055-bib-0050] Hindle, Z. , Chatfield, S.N. , Phillimore, J. , Bentley, M. , Johnson, J. , Cosgrove, C.A. , Ghaem‐Maghami, M. , Sexton, A. *et al*. (2002) Characterization of *Salmonella enterica* derivatives harboring defined *aroC* and *Salmonella* pathogenicity island 2 type III secretion system (*ssaV*) mutations by immunization of healthy volunteers. Infect Immun 70, 3457–3467. 12065485 10.1128/IAI.70.7.3457-3467.2002 PMC128087

[jam15055-bib-0051] Husseiny, M.I. , Wartha, F. and Hensel, M. (2007) Recombinant vaccines based on translocated effector proteins of *Salmonella* pathogenicity island 2. Vaccine 25, 185–193. 16887239 10.1016/j.vaccine.2005.11.020

[jam15055-bib-0052] Islam, S.T. and Lam, J.S. (2014) Synthesis of bacterial polysaccharides via the *Wzx/Wzy*‐dependent pathway. Can J Microbiol 60, 697–716. 25358682 10.1139/cjm-2014-0595

[jam15055-bib-0053] Jorbeck, H.J. , Svenson, S.B. and Lindberg, A.A. (1979) Immunochemistry of *Salmonella* O‐antigens: specificity of rabbit antibodies against the O‐antigen 4 determinant elicited by whole bacteria and O‐antigen 4 specific saccharide‐protein conjugates. J Immunol 123, 1376–1381. 89168

[jam15055-bib-0054] Jorbeck, H.J. , Svenson, S.B. and Lindberg, A.A. (1981) Artificial *Salmonella* vaccines: *Salmonella* Typhimurium O‐antigen‐specific oligosaccharide‐protein conjugates elicit opsonizing antibodies that enhance phagocytosis. Infect Immun 32, 497–502. 7019072 10.1128/iai.32.2.497-502.1981 PMC351473

[jam15055-bib-0055] Khan, S. , Chatfield, S. , Stratford, R. , Bedwell, J. , Bentley, M. , Sulsh, S. , Giemza, R. , Smith, S. *et al*. (2007) Ability of SPI2 mutant of *S*. *typhi* to effectively induce antibody responses to the mucosal antigen enterotoxigenic *E. coli* heat labile toxin B subunit after oral delivery to humans. Vaccine 25, 4175–4182. 17412462 10.1016/j.vaccine.2007.03.007 PMC2652036

[jam15055-bib-0056] Kingsley, R.A. , van Amsterdam, K. , Kramer, N. and Baumler, A.J. (2000) The *shdA* gene is restricted to serotypes of *Salmonella enterica* subspecies I and contributes to efficient and prolonged fecal shedding. Infect Immun 68, 2720–2727. 10768965 10.1128/iai.68.5.2720-2727.2000 PMC97480

[jam15055-bib-0057] Kingsley, R.A. , Humphries, A.D. , Weening, E.H. , De Zoete, M.R. , Winter, S. , Papaconstantinopoulou, A. , Dougan, G. and Baumler, A.J. (2003) Molecular and phenotypic analysis of the CS54 island of *Salmonella enterica* serotype Typhimurium: identification of intestinal colonization and persistence determinants. Infect Immun 71, 629–640. 12540539 10.1128/IAI.71.2.629-640.2003 PMC145368

[jam15055-bib-0058] Kingsley, R.A. , Keestra, A.M. , de Zoete, M.R. and Baumler, A.J. (2004) The ShdA adhesin binds to the cationic cradle of the fibronectin 13FnIII repeat module: evidence for molecular mimicry of heparin binding. Mol Microbiol 52, 345–355. 15066025 10.1111/j.1365-2958.2004.03995.x

[jam15055-bib-0059] Kingsley, R.A. , Santos, R.L. , Keestra, A.M. , Adams, L.G. and Baumler, A.J. (2002) *Salmonella enterica* serotype Typhimurium ShdA is an outer membrane fibronectin‐binding protein that is expressed in the intestine. Mol Microbiol 43, 895–905. 11929540 10.1046/j.1365-2958.2002.02805.x

[jam15055-bib-0060] Kirkpatrick, B.D. , McKenzie, R. , O'Neill, J.P. , Larsson, C.J. , Bourgeois, A.L. , Shimko, J. , Bentley, M. , Makin, J. *et al*. (2006) Evaluation of *Salmonella enterica* serovar Typhi (Ty2 *aroC‐ssaV‐*) M01ZH09, with a defined mutation in the *Salmonella* pathogenicity island 2, as a live, oral typhoid vaccine in human volunteers. Vaccine 24, 116–123. 16140433 10.1016/j.vaccine.2005.08.008

[jam15055-bib-0061] Konadu, E.Y. , Lin, F.Y. , Ho, V.A. , Thuy, N.T. , Van Bay, P. , Thanh, T.C. , Khiem, H.B. , Trach, D.D. *et al*. (2000) Phase 1 and phase 2 studies of *Salmonella enterica* serovar Paratyphi A O‐specific polysaccharide‐tetanus toxoid conjugates in adults, teenagers, and 2‐ to 4‐year‐old children in Vietnam. Infect Immun 68, 1529–1534. 10678970 10.1128/iai.68.3.1529-1534.2000 PMC97311

[jam15055-bib-0062] Kong, W. , Wanda, S.Y. , Zhang, X. , Bollen, W. , Tinge, S.A. , Roland, K.L. and Curtiss, R. III (2008) Regulated programmed lysis of recombinant *Salmonella* in host tissues to release protective antigens and confer biological containment. Proc Natl Acad Sci U S A 105, 9361–9366. 18607005 10.1073/pnas.0803801105 PMC2453710

[jam15055-bib-0063] Kopecko, D.J. , Sieber, H. , Ures, J.A. , Furer, A. , Schlup, J. , Knof, U. , Collioud, A. , Xu, D. *et al*. (2009) Genetic stability of vaccine strain *Salmonella* Typhi Ty21a over 25 years. Int J Med Microbiol 299, 233–246. 19121604 10.1016/j.ijmm.2008.09.003

[jam15055-bib-0064] Kotton, C.N. , Lankowski, A.J. , Scott, N. , Sisul, D. , Chen, L.M. , Raschke, K. , Borders, G. , Boaz, M. *et al*. (2006) Safety and immunogenicity of attenuated *Salmonella enterica* serovar Typhimurium delivering an HIV‐1 Gag antigen via the *Salmonella* type III secretion system. Vaccine 24, 6216–6224. 16824652 10.1016/j.vaccine.2006.05.094

[jam15055-bib-0065] Launay, O. , Lewis, D.J.M. , Anemona, A. , Loulergue, P. , Leahy, J. , Scire, A.S. , Maugard, A. , Marchetti, E. *et al*. (2017) Safety profile and immunologic responses of a novel vaccine against *Shigella sonnei* administered intramuscularly, intradermally and intranasally: results from two parallel randomized phase 1 clinical studies in healthy adult volunteers in Europe. EBioMedicine 22, 164–172. 28735965 10.1016/j.ebiom.2017.07.013 PMC5552227

[jam15055-bib-0066] Launay, O. , Ndiaye, A.G.W. , Conti, V. , Loulergue, P. , Scire, A.S. , Landre, A.M. , Ferruzzi, P. , Nedjaai, N. *et al*. (2019) Booster vaccination with GVGH *Shigella sonnei* 1790GAHB GMMA vaccine compared to single vaccination in unvaccinated healthy European adults: results from a phase 1 clinical trial. Front Immunol 10, 335. 30906291 10.3389/fimmu.2019.00335 PMC6418009

[jam15055-bib-0067] Levine, M.M. , Ferreccio, C. , Black, R.E. , Lagos, R. , San Martin, O. and Blackwelder, W.C. (2007) Ty21a live oral typhoid vaccine and prevention of paratyphoid fever caused by *Salmonella enterica* serovar Paratyphi B. Clin Infect Dis 45(Suppl 1), S24–28. 17582564 10.1086/518141

[jam15055-bib-0068] Levine, M.M. , Tacket, C.O. and Sztein, M.B. (2001) Host‐*Salmonella* interaction: human trials. Microbes Infect 3, 1271–1279. 11755415 10.1016/s1286-4579(01)01487-3

[jam15055-bib-0069] Majowicz, S.E. , Musto, J. , Scallan, E. , Angulo, F.J. , Kirk, M. , O'Brien, S.J. , Jones, T.F. , Fazil, A. *et al*. (2019) The global burden of non‐typhoidal *Salmonella* invasive disease: a systematic analysis for the global burden of disease study 2017. Lancet Infect Dis 19, 1312–1324. 31562022 10.1016/S1473-3099(19)30418-9 PMC6892270

[jam15055-bib-0070] Mancini, F. , Rossi, O. , Necchi, F. and Micoli, F. (2020) OMV vaccines and the role of TLR agonists in immune response. Int J Mol Sci 21, 4416. 10.3390/ijms21124416 PMC7352230 32575921

[jam15055-bib-0071] Mastroeni, P. , Chabalgoity, J.A. , Dunstan, S.J. , Maskell, D.J. and Dougan, G. (2001) *Salmonella*: immune responses and vaccines. Vet J 161, 132–164. 11243685 10.1053/tvjl.2000.0502

[jam15055-bib-0072] Matsui, H. , Suzuki, M. , Isshiki, Y. , Kodama, C. , Eguchi, M. , Kikuchi, Y. , Motokawa, K. , Takaya, A. *et al*. (2003) Oral immunization with ATP‐dependent protease‐deficient mutants protects mice against subsequent oral challenge with virulent *Salmonella enterica* serovar Typhimurium. Infect Immun 71, 30–39. 12496146 10.1128/IAI.71.1.30-39.2003 PMC143154

[jam15055-bib-0073] Metzger, W.G. , Mansouri, E. , Kronawitter, M. , Diescher, S. , Soerensen, M. , Hurwitz, R. , Bumann, D. , Aebischer, T. *et al*. (2004) Impact of vector‐priming on the immunogenicity of a live recombinant *Salmonella enterica* serovar Typhi Ty21a vaccine expressing urease A and B from *Helicobacter pylori* in human volunteers. Vaccine 22, 2273–2277. 15149786 10.1016/j.vaccine.2003.11.020

[jam15055-bib-0074] Micoli, F. , Rondini, S. , Alfini, R. , Lanzilao, L. , Necchi, F. , Negrea, A. , Rossi, O. , Brandt, C. *et al*. (2018) Comparative immunogenicity and efficacy of equivalent outer membrane vesicle and glycoconjugate vaccines against nontyphoidal *Salmonella* . Proc Natl Acad Sci U S A 115, 10428–10433. 30262653 10.1073/pnas.1807655115 PMC6187145

[jam15055-bib-0075] Miletic, S. , Goessweiner‐Mohr, N. and Marlovits, T.C. (2020) The structure of the type III secretion system needle complex. Curr Top Microbiol Immunol 427, 67–90. 31667599 10.1007/82_2019_178

[jam15055-bib-0076] Mogasale, V. , Maskery, B. , Ochiai, R.L. , Lee, J.S. , Mogasale, V.V. , Ramani, E. , Kim, Y.E. , Park, J.K. *et al*. (2014) Burden of typhoid fever in low‐income and middle‐income countries: a systematic, literature‐based update with risk‐factor adjustment. Lancet Glob Health 2, e570–e580. 25304633 10.1016/S2214-109X(14)70301-8

[jam15055-bib-0077] Murray, G.L. , Attridge, S.R. and Morona, R. (2003) Regulation of *Salmonella* Typhimurium lipopolysaccharide O antigen chain length is required for virulence: identification of FepE as a second Wzz. Mol Microbiol 47, 1395–1406. 12603743 10.1046/j.1365-2958.2003.03383.x

[jam15055-bib-0078] Needham, B.D. , Carroll, S.M. , Giles, D.K. , Georgiou, G. , Whiteley, M. and Trent, M.S. (2013) Modulating the innate immune response by combinatorial engineering of endotoxin. Proc Natl Acad Sci USA 110, 1464–1469. 23297218 10.1073/pnas.1218080110 PMC3557076

[jam15055-bib-0079] Obiero, C.W. , Ndiaye, A.G.W. , Scire, A.S. , Kaunyangi, B.M. , Marchetti, E. , Gone, A.M. , Schutte, L.D. , Riccucci, D. *et al*. (2017) A phase 2a randomized study to evaluate the safety and immunogenicity of the 1790GAHB generalized modules for membrane antigen vaccine against *Shigella sonnei* administered intramuscularly to adults from a shigellosis‐endemic country. Front Immunol 8, 1884. 29375556 10.3389/fimmu.2017.01884 PMC5763125

[jam15055-bib-0080] Osorio, M. , Wu, Y. , Singh, S. , Merkel, T.J. , Bhattacharyya, S. , Blake, M.S. and Kopecko, D.J. (2009) Anthrax protective antigen delivered by *Salmonella enterica* serovar Typhi Ty21a protects mice from a lethal anthrax spore challenge. Infect Immun 77, 1475–1482. 19179420 10.1128/IAI.00828-08 PMC2663156

[jam15055-bib-0081] Pasetti, M.F. , Pickett, T.E. , Levine, M.M. and Sztein, M.B. (2000) A comparison of immunogenicity and in vivo distribution of *Salmonella enterica* serovar Typhi and Typhimurium live vector vaccines delivered by mucosal routes in the murine model. Vaccine 18, 3208–3213. 10869765 10.1016/s0264-410x(00)00142-0

[jam15055-bib-0082] Pickett, T.E. , Pasetti, M.F. , Galen, J.E. , Sztein, M.B. and Levine, M.M. (2000) In vivo characterization of the murine intranasal model for assessing the immunogenicity of attenuated *Salmonella enterica* serovar Typhi strains as live mucosal vaccines and as live vectors. Infect Immun 68, 205–213. 10603389 10.1128/iai.68.1.205-213.2000 PMC97122

[jam15055-bib-0083] Pinaud, L. , Sansonetti, P.J. and Phalipon, A. (2018) Host cell targeting by enteropathogenic bacteria T3SS effectors. Trends Microbiol 26, 266–283. 29477730 10.1016/j.tim.2018.01.010

[jam15055-bib-0084] van der Pol, L. , Stork, M. and van der Ley, P. (2015) Outer membrane vesicles as platform vaccine technology. Biotechnol J 10, 1689–1706. 26912077 10.1002/biot.201400395 PMC4768646

[jam15055-bib-0085] Ramirez, K. , Ditamo, Y. , Galen, J.E. , Baillie, L.W. and Pasetti, M.F. (2010) Mucosal priming of newborn mice with *S*. Typhi Ty21a expressing anthrax protective antigen (PA) followed by parenteral PA‐boost induces B and T cell‐mediated immunity that protects against infection bypassing maternal antibodies. Vaccine 28, 6065–6075. 20619377 10.1016/j.vaccine.2010.06.089 PMC2938045

[jam15055-bib-0086] Rappuoli, R. (2018) Glycoconjugate vaccines: principles and mechanisms. Sci Transl Med 10, eaat4615. 30158151 10.1126/scitranslmed.aat4615

[jam15055-bib-0087] Roland, K.L. and Brenneman, K.E. (2013) *Salmonella* as a vaccine delivery vehicle. Expert Rev Vaccines 12, 1033–1045. 24053397 10.1586/14760584.2013.825454 PMC3956298

[jam15055-bib-0088] Rosenqvist, E. , Hoiby, E.A. , Bjune, G. , Aase, A. , Halstensen, A. , Lehmann, A.K. , Paulssen, J. , Holst, J. *et al*. (1998) Effect of aluminium hydroxide and meningococcal serogroup C capsular polysaccharide on the immunogenicity and reactogenicity of a group B *Neisseria meningitidis* outer membrane vesicle vaccine. Dev Biol Stand 92, 323–333. 9554288

[jam15055-bib-0089] Rossi, O. , Caboni, M. , Negrea, A. , Necchi, F. , Alfini, R. , Micoli, F. , Saul, A. , MacLennan, C.A. *et al*. (2016) Toll‐like receptor activation by generalized modules for membrane antigens from lipid A mutants of *Salmonella enterica* serovars Typhimurium and Enteritidis. Clin Vaccine Immunol 23, 304–314. 26865597 10.1128/CVI.00023-16 PMC4820502

[jam15055-bib-0090] Schetters, S.T.T. , Jong, W.S.P. , Horrevorts, S.K. , Kruijssen, L.J.W. , Engels, S. , Stolk, D. , Daleke‐Schermerhorn, M.H. , Garcia‐Vallejo, J. *et al*. (2019) Outer membrane vesicles engineered to express membrane‐bound antigen program dendritic cells for cross‐presentation to CD8(+) T cells. Acta Biomater 91, 248–257. 31003032 10.1016/j.actbio.2019.04.033

[jam15055-bib-0091] Schuster, O. , Sears, K.T. , Ramachandran, G. , Fuche, F.J. , Curtis, B. , Tennant, S.M. and Simon, R. (2019) Immunogenicity and protective efficacy against *Salmonella* C2–C3 infection in mice immunized with a glycoconjugate of *S*. Newport Core‐O polysaccharide linked to the homologous serovar FliC protein. Hum Vaccin Immunother 15, 1436–1444. 29873578 10.1080/21645515.2018.1483808 PMC6663135

[jam15055-bib-0092] Schwechheimer, C. and Kuehn, M.J. (2015) Outer‐membrane vesicles from Gram‐negative bacteria: biogenesis and functions. Nat Rev Microbiol 13, 605–619. 26373371 10.1038/nrmicro3525 PMC5308417

[jam15055-bib-0093] Shakya, M. , Colin‐Jones, R. , Theiss‐Nyland, K. , Voysey, M. , Pant, D. , Smith, N. , Liu, X. , Tonks, S. *et al*. (2019) Phase 3 efficacy analysis of a typhoid conjugate vaccine trial in Nepal. N Engl J Med 381, 2209–2218. 31800986 10.1056/NEJMoa1905047 PMC6785806

[jam15055-bib-0094] Sim, B.K.L. , Li, M. , Osorio, M. , Wu, Y. , Wai, T.T. , Peterson, J.W. , James, E.R. , Chakravarty, S. *et al*. (2017) Protection against inhalation anthrax by immunization with *Salmonella enterica* serovar Typhi Ty21a stably producing protective antigen of *Bacillus anthracis* . NPJ Vaccines 2, 17. 29263873 10.1038/s41541-017-0018-4 PMC5627300

[jam15055-bib-0095] Simanjuntak, C.H. , Totosudirjo, H. , Haryanto, P. , Suprijanto, E. , Paleologo, F.P. , Punjabi, N.H. , Witham, N.D. , Darmowigoto, R. *et al*. (1991) Oral immunisation against typhoid fever in Indonesia with Ty21a vaccine. Lancet 338, 1055–1059. 1681365 10.1016/0140-6736(91)91910-m

[jam15055-bib-0096] Simon, R. , Curtis, B. , Deumic, V. , Nicki, J. , Tennant, S.M. , Pasetti, M.F. , Lees, A. , Wills, P.W. *et al*. (2014) A scalable method for biochemical purification of *Salmonella* flagellin. Protein Expr Purif 102, 1–7. 25050462 10.1016/j.pep.2014.07.005 PMC4175188

[jam15055-bib-0097] Simon, R. , Tennant, S.M. , Wang, J.Y. , Schmidlein, P.J. , Lees, A. , Ernst, R.K. , Pasetti, M.F. , Galen, J.E. *et al*. (2011) *Salmonella enterica* serovar Enteritidis core O polysaccharide conjugated to H:g, m flagellin as a candidate vaccine for protection against invasive infection with S. enteritidis. Infect Immun 79, 4240–4249. 21807909 10.1128/IAI.05484-11 PMC3187246

[jam15055-bib-0098] Sztein, M.B. (2007) Cell‐mediated immunity and antibody responses elicited by attenuated *Salmonella enterica* serovar Typhi strains used as live oral vaccines in humans. Clin Infect Dis 45(Suppl 1), S15–S19. 17582562 10.1086/518140

[jam15055-bib-0099] Tacket, C.O. and Levine, M.M. (2007) CVD 908, CVD 908‐htrA, and CVD 909 live oral typhoid vaccines: a logical progression. Clin Infect Dis 45(Suppl 1), S20–23. 17582563 10.1086/518135

[jam15055-bib-0100] Tacket, C.O. , Pasetti, M.F. , Sztein, M.B. , Livio, S. and Levine, M.M. (2004) Immune responses to an oral typhoid vaccine strain that is modified to constitutively express Vi capsular polysaccharide. J Infect Dis 190, 565–570. 15243933 10.1086/421469

[jam15055-bib-0101] Tennant, S.M. and Levine, M.M. (2015) Live attenuated vaccines for invasive *Salmonella* infections. Vaccine 33(Suppl. 3), C36–C41. 25902362 10.1016/j.vaccine.2015.04.029 PMC4469493

[jam15055-bib-0102] Tennant, S.M. , Schmidlein, P. , Simon, R. , Pasetti, M.F. , Galen, J.E. and Levine, M.M. (2015) Refined live attenuated *Salmonella enterica* serovar Typhimurium and Enteritidis vaccines mediate homologous and heterologous serogroup protection in mice. Infect Immun 83, 4504–4512. 26351285 10.1128/IAI.00924-15 PMC4645371

[jam15055-bib-0103] Tennant, S.M. , Wang, J.Y. , Galen, J.E. , Simon, R. , Pasetti, M.F. , Gat, O. and Levine, M.M. (2011) Engineering and preclinical evaluation of attenuated nontyphoidal *Salmonella* strains serving as live oral vaccines and as reagent strains. Infect Immun 79, 4175–4185. 21807911 10.1128/IAI.05278-11 PMC3187273

[jam15055-bib-0104] Torok, I. and Kari, C. (1980) Accumulation of ppGpp in a relA mutant of *Escherichia coli* during amino acid starvation. J Biol Chem 255, 3838–3840. 6768741

[jam15055-bib-0105] Tramont, E.C. , Chung, R. , Berman, S. , Keren, D. , Kapfer, C. and Formal, S.B. (1984) Safety and antigenicity of typhoid‐*Shigella sonnei* vaccine (strain 5076–1C). J Infect Dis 149, 133–136. 6366077 10.1093/infdis/149.2.133

[jam15055-bib-0106] Vindurampulle, C.J. , Cuberos, L.F. , Barry, E.M. , Pasetti, M.F. and Levine, M.M. (2004) Recombinant *Salmonella enterica* serovar Typhi in a prime‐boost strategy. Vaccine 22, 3744–3750. 15315855 10.1016/j.vaccine.2004.03.025

[jam15055-bib-0107] Wang, J.Y. , Noriega, F.R. , Galen, J.E. , Barry, E. and Levine, M.M. (2000) Constitutive expression of the Vi polysaccharide capsular antigen in attenuated *Salmonella enterica* serovar Typhi oral vaccine strain CVD 909. Infect Immun 68, 4647–4652. 10899868 10.1128/iai.68.8.4647-4652.2000 PMC98400

[jam15055-bib-0108] Watson, D.C. , Robbins, J.B. and Szu, S.C. (1992) Protection of mice against *Salmonella* Typhimurium with an O‐specific polysaccharide‐protein conjugate vaccine. Infect Immun 60, 4679–4686. 1383154 10.1128/iai.60.11.4679-4686.1992 PMC258218

[jam15055-bib-0109] Whitfield, C. (2006) Biosynthesis and assembly of capsular polysaccharides in *Escherichia coli* . Annu Rev Biochem 75, 39–68. 16756484 10.1146/annurev.biochem.75.103004.142545

[jam15055-bib-0110] Wu, Y. , Chakravarty, S. , Li, M. , Wai, T.T. , Hoffman, S.L. and Sim, B.K. (2017) Development of a live attenuated bivalent oral vaccine against *Shigella sonnei* shigellosis and typhoid fever. J Infect Dis 215, 259–268. 27803169 10.1093/infdis/jiw528 PMC6516681

[jam15055-bib-0111] Xu, X. , Hegazy, W.A. , Guo, L. , Gao, X. , Courtney, A.N. , Kurbanov, S. , Liu, D. , Tian, G. *et al*. (2014) Effective cancer vaccine platform based on attenuated *Salmonella* and a type III secretion system. Cancer Res 74, 6260–6270. 25213323 10.1158/0008-5472.CAN-14-1169 PMC4216746

[jam15055-bib-0112] Zahn, K. (1996) Overexpression of an mRNA dependent on rare codons inhibits protein synthesis and cell growth. J Bacteriol 178, 2926–2933. 8631683 10.1128/jb.178.10.2926-2933.1996 PMC178030

[jam15055-bib-0113] Zhao, B. and Houry, W.A. (2010) Acid stress response in enteropathogenic gammaproteobacteria: an aptitude for survival. Biochem Cell Biol 88, 301–314. 20453931 10.1139/o09-182

